# The Impact of Linoleic Acid on Infant Health in the Absence or Presence of DHA in Infant Formulas

**DOI:** 10.3390/nu15092187

**Published:** 2023-05-04

**Authors:** Alexandra W. C. Einerhand, Wiola Mi, Alfred Haandrikman, Xiao-Yang Sheng, Philip C. Calder

**Affiliations:** 1 Einerhand Science & Innovation, Nutrition Consultancy, 1815 JN Alkmaar, The Netherlands; 2 Bunge Loders Croklaan Nutrition, Shanghai 200051, China; wiola.mi@bunge.com; 3 Independent Consultant, 3823 HH Amersfoort, The Netherlands; 4 Department of Developmental Behavioral Pediatric & Children Healthcare, Xinhua Hospital, School of Medicine, Shanghai Jiao Tong University, Shanghai 200051, China; 5 School of Human Development and Health, Faculty of Medicine, University of Southampton, Southampton SO16 6YD, UK; 6 NIHR Southampton Biomedical Research Centre, University Hospital Southampton NHS Foundation Trust and University of Southampton, Southampton SO16 6YD, UK

**Keywords:** linoleic acid (LA), α-linolenic acid (ALA), polyunsaturated fatty acids (PUFAs), breast milk composition, infant development, infant formula (IF), lipids, docosahexaenoic acid (DHA)

## Abstract

Both linoleic acid (LA) and α-linolenic acid (ALA) are essential dietary fatty acids, and a balanced dietary supply of these is of the utmost importance for health. In many countries across the globe, the LA level and LA/ALA ratio in breast milk (BM) are high. For infant formula (IF), the maximum LA level set by authorities (e.g., Codex or China) is 1400 mg LA/100 kcal ≈ 28% of total fatty acid (FA) ≈ 12.6% of energy. The aims of this study are: (1) to provide an overview of polyunsaturated fatty acid (PUFA) levels in BM across the world, and (2) to determine the health impact of different LA levels and LA/ALA ratios in IF by reviewing the published literature in the context of the current regulatory framework. The lipid composition of BM from mothers living in 31 different countries was determined based on a literature review. This review also includes data from infant studies (intervention/cohort) on nutritional needs regarding LA and ALA, safety, and biological effects. The impact of various LA/ALA ratios in IF on DHA status was assessed within the context of the current worldwide regulatory framework including China and the EU. Country averages of LA and ALA in BM range from 8.5–26.9% FA and 0.3–2.65% FA, respectively. The average BM LA level across the world, including mainland China, is below the maximum 28% FA, and no toxicological or long-term safety data are available on LA levels > 28% FA. Although recommended IF LA/ALA ratios range from 5:1 to 15:1, ratios closer to 5:1 seem to promote a higher endogenous synthesis of DHA. However, even those infants fed IF with more optimal LA/ALA ratios do not reach the DHA levels observed in breastfed infants, and the levels of DHA present are not sufficient to have positive effects on vision. Current evidence suggests that there is no benefit to going beyond the maximum LA level of 28% FA in IF. To achieve the DHA levels found in BM, the addition of DHA to IF is necessary, which is in line with regulations in China and the EU. Virtually all intervention studies investigating LA levels and safety were conducted in Western countries in the absence of added DHA. Therefore, well-designed intervention trials in infants across the globe are required to obtain clarity about optimal and safe levels of LA and LA/ALA ratios in IF.

## 1. Introduction

Breastfeeding is generally the most effective way to maintain infant health. In cases where breastfeeding is not possible, infant formula (IF) feeding is the appropriate alternative, providing nutrients and sufficient energy to enable infant growth, development and long-term health. BM is composed of various macronutrients, including proteins, carbohydrates and lipids, as well as more minor constituents. Its composition varies throughout lactation and may differ between populations due to regional differences and historical changes. IF, on the other hand, is designed to mimic the composition of BM and has also undergone changes in its macronutrient composition over time [1,2].

Protein concentrations in BM decrease during lactation from an average of 2.5 g/100 mL (3.8 g/100 kcal) for colostrum (1–5 days), 1.7 g/100 mL (2.6 g/100 kcal) for transitional milk and 1.3 g/100 mL (2.0 g/100 kcal) for mature BM. The level slowly decreases to about 1.1 g/100 mL (1.6 g/100 kcal) at 6 months of lactation and tends to remain fairly stable thereafter [3]. Protein concentrations in IF have decreased over the past decades from 4 g/100 kcal in the 1970s to minimally 1.8 g/100 kcal nowadays [4].

The main carbohydrate in BM is lactose, a disaccharide that provides a source of energy for infants. The concentration of lactose in BM remains relatively constant throughout lactation (typically around 6–7 g/100 mL), although variations have been reported in different populations [5]. IFs typically contain lactose as the primary carbohydrate source, although in specific formulas (e.g., lactose-free and hypoallergenic formulas) other carbohydrates may be added. 

The lipids in both BM and IF provide around 50% of the total energy needed for an infant [6]. They are especially important for brain development, as lipids constitute nearly 60% of the dry weight of the human brain [7]. The polyunsaturated fatty acids (PUFAs), linoleic acid (LA; 18:2 *n*-6) and α-linolenic acid (ALA; 18:3 *n*-3) are essential fatty acids (FAs) that must come from the diet, as the human body cannot make them. LA and ALA are the main substrates for the production of the long-chain PUFAs (LCPUFAs), arachidonic acid (ARA; 20:4 *n*-6) and docosahexaenoic acid (DHA; 22:6 *n*-3). About 35% of the lipids in the grey matter of the brain are LCPUFAs, and DHA and ARA are especially important building blocks of the brain [8,9].

PUFAs form a significant portion (15–30%) of the total FA profile present in BM [10]. It is, therefore, of the utmost importance to guarantee that PUFAs can be used in IFs in a safe way with proven efficacy. The composition of BM can provide important guidance for IF composition in the absence of quantitative nutrient requirement data [11]. However, the levels of PUFAs in BM—including LA and ALA—vary and are mainly dependent on the maternal diet. Although ALA levels have remained quite stable, a considerable increase in the LA content of BM in countries such as the US has been observed over the past decades [12]. This correlates with a drastic increase in the global consumption of vegetable oils containing LA as the primary PUFA during the same period [13,14,15]. As a result, a distinct increase in the LA/ALA ratio in BM has been observed, from about 6:1 before 1970 to over 16:1 in 2010 [12,16], potentially reducing the bioconversion of ALA to DHA [17].

A literature review was used in this study to obtain data regarding the FA composition of BM from mothers around the world, especially focusing on DHA, ALA, LA and LA-containing triglycerides. As LA concentrations in BM and diets are determined by region/culture/diet, both Eastern and Western perspectives were taken into account, and potential gaps in knowledge were identified. Scientific data from infant (intervention/cohort) studies on nutritional needs regarding LA, ALA and safety and biological effects of IF with different levels of PUFAs were reviewed. The addition of DHA into IF is mandatory in many countries, but the literature consists almost entirely of studies conducted on IF without added DHA. Therefore, the impact of various LA/ALA ratios in IF on infant DHA status was assessed in the absence of added DHA in the formula. The totality of the data was put into the context of the current recommendations and guidelines regarding IF lipid composition from authorities around the world including Codex Alimentarius (Codex), the European Commission (EC directives) and Chinese GB standards.

The PUFAs in IF can have an important effect on infant health outcomes. This review aims to determine the health impacts of different levels of LA and different ratios of LA/ALA in IF based on the latest science within the context of the current regulatory framework, which requires the addition of DHA to IF in many countries.

## 2. Levels of Main PUFAs in Breast Milk across the World

### 2.1. Levels of Main PUFAs in Breast Milk across Different Countries

**Figure 1:**
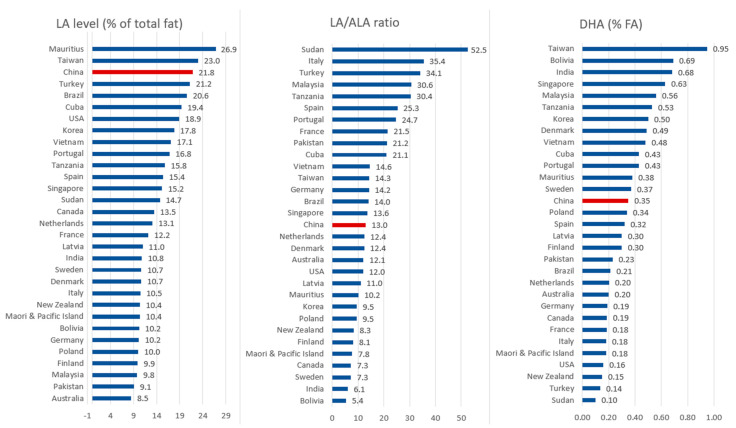
Average LA levels (% FA), LA/ALA ratios and DHA levels (% FA) in breast milk in different countries/regions. For ALA levels (% FA) and references, see Supplemental Information.

In 2019, an extensive review of the literature reported the FA profile of BM, including LA, ALA, DHA and ARA [10]. That analysis included data from 55 studies conducted worldwide and published between 1980 and 2018. Figure 1 presents country averages based on that analysis plus data extracted from 17 additional studies published after 2018 (Supplemental Table S1).

Based on this updated analysis, the country averages for LA and ALA range between 8.5 and 26.9% (Figure 1), and 0.3 and 2.65% of total FAs (Supplemental Table S1), respectively. These levels may vary depending on various maternal, nutritional, lifestyle and genetic factors. As a result, the ratios of LA/ALA also vary between 5 and 53 (Figure 1). It is of note that in about one-third of countries around the world, the LA/ALA ratio is higher than the recommended ratio of 15:1 set by Codex [18,19] and Chinese GB standards (GB 10765-2021, GB 10766-2021 and GB 10767-2021). However, even if the LA level is high, the LA/ALA ratio can sometimes be below 15, as is the case in mainland China (Figure 1, red bars). This is due to the fact that the ALA level is also high (Supplemental Table S1). The DHA levels in BM range between 0.10% FA and 0.95% FA, while mainland China ranked in the middle with a DHA level of 0.35% FA (Figure 1).

### 2.2. Levels of Main PUFAs in Breast Milk across Different Regions in China

Two recent systematic reviews focused on the BM composition of Chinese women [20,21]. One of these reviews was conducted with the specific aim of identifying regional differences in BM FA profiles [20]. It included 21 studies and categorized the regions of China in three different ways: (1) north vs. south; (2) inland vs. coastal and (3) according to socioeconomic development levels (Tiers 1–3).

In China, the BM content of LA was fairly consistent across regions [20]. For LA, the variations were minor, being slightly higher in northern China (22.3% FA) compared to the southern population (21.4% FA), and higher in Tier 1 (22.6% FA) compared to the lower-tier cities (21.4% FA). ALA concentrations varied much more than LA, being highest in the lower compared to the higher-tier cities (1.99% vs. 1.44% FA) and higher in inland China than in coastal China (1.8% vs. 1.41% FA). As a result, the ratio of LA/ALA also varied considerably. The highest ratios were found in Tier 1 cities and coastal regions (16 and 15.5), whereas the lowest ratios were observed in the lowest-tier cities and in inland China (10.8 and 12.1). 

DHA levels varied substantially across the various regions but were overall in the middle range among countries in the world (Supplemental Table S1 and Figure 1). DHA was much lower in northern China compared to the south (0.32% vs. 0.38% FA). Similarly, DHA was lower in inland China compared to the coastal areas (0.34% vs. 0.37% FA). Lower-tier cities had slightly higher DHA concentrations compared to higher-tier cities (0.36% vs. 0.35% FA) [20]. Different diets are most likely the main driver behind the noted variations [22], but the conversion rate of LA and ALA into ARA and DHA, respectively, can vary among mothers too. For example, a higher LA level could inhibit the conversion of ALA to DHA [18], but the impact is very limited. Sun et al. did not observe an association between LA and DHA [20]. In addition, conversion rates of ALA to DHA in adults are estimated to be fairly low (0.5–9%) [23]. Therefore, differences in diet remain the most likely explanation for the noted differences in DHA [20].

## 3. Levels of Special LA-Containing Triglycerides in BM

Lipids in BM are generally present in the form of triglycerides (TGs), each consisting of three FAs esterified onto a glycerol backbone. The principal TGs in mature BM are 1,3-dioleoyl-2-palmitoylglycerol (OPO) and 1-oleoyl-2-palmitoyl-3-linoleoylglycerol (OPL). These TGs have a unique stereospecific structure (e.g., OPO (oleic-palmitic-oleic) or OPL (oleic-palmitic-linoleic)), with palmitic acid primarily esterified at the sn-2 position (∼60–75%) and unsaturated fatty acids mainly at the sn-1,3 positions [24]. TGs with most of their palmitic acid at the sn-2 position have sometimes been called β-palmitate.

The main forms of TGs in mature BM of Western mothers are OPO (9.4–29%) and OPL (3.0–20%). OPO and OPL are also the major forms in Chinese BM, but in this case, OPL (5.8–28%) is often more abundantly present than OPO (3.2–19.5%) [25,26,27,28]. The phenomenon of a higher OPL/OPO ratio in Chinese versus Western BM can be explained by either an elevated LA content or a lower oleic acid (OA) content. However, the OA content reported in Chinese BM [29,30] was very similar to that seen in most Western countries [31,32]. Therefore, the high LA content in Chinese BM might be most likely the main reason for its high OPL/OPO ratio.

The digestion/absorption and clinical benefits of OPO from a vegetable source have been extensively studied in full-term and premature infants [33,34,35]. However, little is known about the effects of OPL. Only recently (2021), an in vitro study showed that OPL had a lower digestibility but higher absorptivity compared with OPO [36], but how this translates into an in vivo situation is unclear. More research is needed to clarify the effects of OPL versus OPO.

## 4. Changing LA Levels in BM over Time and the Influence of Diet

### 4.1. The Level of LA in BM Has Increased over the Past Decades

LA is the primary PUFA in numerous vegetable oils [15]. As a result, BM from vegetarian women has a higher concentration of LA than that from omnivores [37]. In the last 50 years, there has been a steady increase in maternal intake of vegetable oils [13], which is reflected in a substantial increase in the LA content of BM from mothers in the US among other Western countries such as the UK, where the increase was less obvious than in the US [12]. 

In China, this also seems to be the case. The LA level in Chinese mothers’ BM was reported to be 17% FA in 1985, between 18.5% and 20.5% FA in 1995, and by 2020 had increased to levels between 21.4% and 22.6% FA depending on the region [20,38,39]. The consumption of vegetable oils in Chinese cities has significantly increased due to the growing economy and rising incomes [40]. As a result, the LA/ALA ratio in BM is higher in the Tier 1 cities compared to the lower-tier cities and is also higher in coastal areas compared to inland China [20].

### 4.2. The Influence of Diet on PUFA Levels in BM

The effect of diet on BM FA composition was analyzed in a recent large Canadian cohort study (*n* = 1094) [41]. Fish oil supplementation and fatty fish intake were positively associated with DHA and some other *n*-3 PUFAs such as EPA and DPA. A higher Healthy Eating Index 2010 score (HEI-2010)—reflecting diet quality—was associated with higher LA, higher total *n*-6 PUFAs and slightly higher total *n*-3 PUFAs (in particular DHA), as well as lower individual and total saturated FAs (SFAs). The same study also showed that LA in BM positively correlated to total and individual *n*-3 PUFAs, and negatively correlated to total and individual SFAs and palmitic acid [41].

In China, several studies focused on the effect of the maternal diet on the BM FA content during lactation [22,29,42,43,44]. The most recent one was carried out by Wu et al. in Beijing [29]. This study revealed excessive fat intake amongst more than 75% of the lactating women who participated in the study compared with the dietary reference intakes for lactating women recommended in the 2016 Dietary Guidelines for Chinese Residents. Compared to the recommended intake, 55.8% of participating mothers had an excessive nut intake, and 40.4% had an excessive intake of cooking oils, which is likely to be a main contributor to the high-fat consumption of the lactating women. This high fat intake is in line with the results of two studies conducted in southeast and northeast China [44,45]. However, the study conducted by Wu et al. concerns a small pilot study (*n* = 52) performed in one city in China; future studies in different areas across China need to determine the validity of these findings. Studies from other regions, such as Spain, the UK, the USA, Brazil and another area of China showed little change in the maternal dietary pattern from preconception to the postpartum period [45,46,47,48,49]. 

The Beijing study by Wu et al. demonstrated that LA accounted for 23.9% of the total FAs, similar to the concentrations reported earlier [20,29]. LA content was positively correlated with the intake of soybeans and soybean products, whereas a negative correlation was identified with seafood consumption. It was previously shown by Liu et al. that a higher consumption of vegetable oils was the main explanation for the higher concentration of LA in BM in China [43]. To demonstrate this, Liu et al. investigated the FA composition of BM and the related diets of mothers in five different regions of China: Shandong, Changchun, Chongqing, Guangzhou and Hohhot [43]. The mean LA, ALA and DHA levels of mature BM (LA: 23.3% FA; ALA: 1.7% FA; DHA: 0.31% FA) across these five regions were similar to the ones reported in Section 2.2 of this review Klik of tik om tekst in te voeren. In addition, the average DHA level of mature BM was in the middle compared with other countries, but the average LA content was significantly higher compared with other countries. This is fully in line with the levels reported in Section 2.2. The DHA contents in colostrum in Shandong (0.67% FA) and Guangzhou (0.60% FA) are the highest and those in Hohhot the lowest (0.39% FA). This may be related to the geographical location of Shandong and Guangzhou being near the ocean where there is an abundance of seafood, which is especially rich in DHA.

The LA content of mature BM in Changchun was the highest (30.8% FA), and that in Chongqing was the lowest (15.7% FA). During lactation, the LA content continuously increased in four of the five regions, the exception being Hohhot. The mean ALA level of mature BM among the five regions was the highest in Changchun (2.1% FA), and the lowest in Guangzhou (0.6% FA). The high level of LA and ALA corresponded with high consumption of soybean oil (in line with pilot study results mentioned above) [29], sunflower oil and peanut oil, all of which are rich in LA and ALA. This study also demonstrated that the LA/ALA ratio declined during lactation, but in Shandong and Guangzhou it remained higher than the ratio recommended by Codex and the Chinese GB standards (Section 6). According to Liu et al., the *n*-6/*n*-3 FA ratio often revealed an imbalance due to high consumption of *n*-6 FAs and low intake of *n*-3 FAs, and this imbalance could be reflected in BM [43]. Based on this comprehensive study, Liu et al., recommend that pregnant and lactating women should eat seafood rich in DHA and consume less vegetable oil to reduce the levels of LA in their BM. Furthermore, Chinese women should be educated on healthy eating during lactation to help avoid the common misconception among Chinese women that they should eat more fatty foods during lactation [43].

## 5. Role of LA and ALA in Infancy

### 5.1. Metabolism of LA and ALA

BM is the primary source of LA and ALA for infants. Around 30% of the LA present in BM is directly transferred from the mothers’ diet, while the rest comes from body stores accumulated by the mother, mainly in fat tissue [50].

In the infant, LA is deposited in body stores, used for LCPUFA synthesis and converted into CO_2_. The relative dietary supply of LA and ALA is of importance for the endogenous synthesis of *n*-6 and *n*-3 LCPUFAs, respectively, because these two precursor FAs compete for desaturases and elongases in the PUFA conversion pathway [11,15]. Studies conducted over 20 years ago on preterm and term infants already showed that both LA and ALA can be converted into LCPUFAs [51,52]. A high ratio of LA/ALA leads to a greater conversion of LA to other *n*-6 PUFAs such as ARA, whereas a low ratio leads to a higher conversion of ALA into other *n*-3 PUFAs, EPA and DHA [53]. As discussed in more detail in Section 6, current IF guidelines recommend avoiding an exceptionally high LA/ALA ratio because it reduces ALA conversion to *n*-3 LCPUFAs. Furthermore, very high levels of LA in formulas, above the recommended levels, may promote LA conversion into ARA and oxygenated metabolites having pro-inflammatory functions. This may result in potentially undesirable effects on allergy, immunity, cognitive and metabolic health, which has been extensively reviewed [11].

### 5.2. Preclinical Studies and Studies in Adults with LA and ALA

Recently, a comprehensive review has been written by an international expert group presenting the preclinical evidence and information from cohort studies [11]. Based on mainly preclinical evidence, the aforementioned group postulated that a disproportionally high LA consumption in infants (in the absence of DHA intake) may reduce *n*-3 LCPUFA synthesis and/or accretion, resulting in a lower DHA status [11]. In addition, the synthesis of *n*-6 LCPUFA—derived pro-inflammatory eicosanoids and adipogenic cytokines—might be increased, with a possible effect on the development and performance of the brain, immune system, fat tissue and other organs [11]. However, preclinical evidence cannot provide definitive proof that the effects actually occur in infants. High LA in BM might be associated with poor neurocognitive outcomes and an increased risk for atopic eczema, allergies and obesity, although there is to date no direct clinical evidence in infants supporting any causal relationship [11].

In adults, there is virtually no scientific evidence available to show that addition of LA to the diet increases the blood concentration of inflammatory markers [54,55]. This conclusion is based on a systematic review of 17 randomized controlled trials (RCTs) and a meta-analysis of 30 RCTs [54,55]. This lack of effect on pro-inflammatory biomarkers might be due to the fact that raising dietary LA levels up to six-fold in adults consuming a Western-type diet has been shown to have no significant effect on plasma ARA levels [53]. New research suggests that LA may even have some positive effects on adult health [56]. Results showed that supplementing the diet with LA enhanced the blood concentration of adiponectin, an adipose tissue hormone that helps cells use blood glucose more effectively. LA also enhanced the concentration of certain oxylipins, a family of oxygenated compounds produced from PUFAs that may play a role in mitigating cardiovascular disease. These findings suggest LA deserves greater scrutiny in adults. However, it is not clear whether these findings can be extrapolated to infants.

The next section focuses on the clinical evidence regarding the effects of LA levels and LA/ALA ratios in IF on infants in order to assess the appropriate levels of LA in IF within the context of the current IF market (with a special focus on China). As the appropriate LA/ALA ratio cannot be assessed without looking at the impact it has on DHA, ALA and ARA, these effects will be mentioned whenever relevant, but a comprehensive description of their health effects is beyond the scope of this review.

### 5.3. Intervention Studies with LA and ALA in Infants

#### 5.3.1. Determination of the Minimum and Maximum Levels for LA Intake

The requirements for LA in IF may best be depicted as a range rather than a single value because of the individual variability in FA status at birth. Going beyond the margins of this range increases the risk of negative effects on infant metabolism, physiological functions and short- and long-term health [11]. In this section, the scientific data from (intervention and cohort) studies in infants on requirements for LA, safety and biological effects are reviewed. 

A literature search in PubMed was carried out that initially identified 54 potentially relevant publications focusing on LA interventions in infants. However, only 20 studies were selected for further analysis. Ten of the 54 papers were not relevant because they reported only the FA composition of BM. However, these papers were analyzed in the context of Section 2 and Section 3. The other papers were excluded because they were not in English, not relevant, reviews, preclinical or methodological studies. Unfortunately, the current number of LA intervention studies in infants is limited, and often from decades ago. These studies are summarized in Table 1, Table 2, Table 3 and Table 4.

The minimum requirement for LA is mainly based on studies conducted by Hansen et al. in the 1950s and 1960s [57,58,59]. For instance, in 1963, Hansen et al. investigated the effects of feeding different IFs with very low LA levels [59] (Table 1). LA deficiency developed in infants who received either a diet practically devoid of fat or one providing 42% of the calories as fat but extremely low in LA (<0.1% of energy (EN)). Manifestations of the deficiency state disappeared when 1% or more of EN as LA was provided. A characteristic symptom of the deficiency was dryness of the skin with desquamation, thickening and later intertrigo. This could be because LA, when incorporated into skin ceramides, is essential for maintaining the water-permeability barrier of the skin, thereby avoiding unnecessary trans-epidermal water loss and associated energy loss from water evaporation [60]. Growth was less in infants with low LA intakes (<1% EN), whereas growth was normal in infants who received 1.3 to 7.3% EN as LA. Hansen’s studies further suggested that an optimal consumption could well be ~4% EN, comparable to the LA content of BM of mothers eating the average American diet in the 1960s.

**Table 1:** LA intervention studies to determine the minimum and maximum levels for LA intake in the absence of DHA.

Author [Reference]	Tested Products	Study Population	Main Findings
Hansen et al. [57], Wiese et al. [58], Hansen et al. [59]	Five different IFs varying in content up to 7.3% EN LA	Term infants at birth (*n* = 428)	LA deficiency developed in infants who received a diet low in LA (<0.1% EN). Manifestations of the deficiency state disappeared when LA was given in IF at ≥1% EN. Deficiency symptoms: dryness of the skin with desquamation, thickening and later intertrigo. Growth was less in infants with low LA intakes, whereas it was normal in infants who received 1.3 to 7.3% EN LA
Naismith et al. [61]	IF with 0.55% EN LA vs. breastfed (BF) reference	Term infants (*n* = 40; *n* = 20 per group)	Growth in length and weight during the first 3 months of life were similar to BF infants. Voluntary food intakes followed the normal pattern. Clinical signs of deficiency were not observed, suggesting that the requirement for LA is less than was formerly believed (i.e., <0.55% EN)
Widdowson et al. [62]	Dutch IF with 58% LA, LA/ALA = 36:1 and British IF with 2% LA, LA/ALA = 2:1 vs. BM control with 8% LA, LA/ALA = 2.5:1	Healthy term infants; 41 British infants; 37 Dutch infants; 2 BF infants	British babies fed IF never had more than 2% of LA in their body fat, and BF babies, 3–4%. In contrast, by 6 weeks the subcutaneous fat of Dutch infants had 25% FA as LA, and by 12 weeks, 46%. No obvious adverse effects were observed in infants raised on IF with close to 60% LA of total fat.
Putnam et al. [63]	Test IF with 45% LA compared to 14% LA in standard IF vs. BM control with 8.8% LA	Heathy term infants; *n* = 16 (Test IF); *n* = 15 (Standard IF); *n* = 9 (BM)	Concentrations of PUFAs in erythrocyte membranes of standard and test IF-fed infants were similar despite very significant differences in the amount of dietary LA. No obvious adverse effects were observed in infants raised on IF with close to 45% FA as LA.

Although this research provided important data on minimum requirements for LA, it was performed using formulas with low fat contents and devoid of both ALA and LCPUFAs. These formulas are not comparable to the IFs currently on the market. On the basis of the aforementioned studies in infants [57,58,59] and some preclinical studies in rats [64], the minimum requirement for LA was extended to 2.7% EN in infants to make sure their dietary requirements would be met [18]. More recently, however, a study in rats demonstrated that LA requirements in the presence of ALA are 1–1.5% EN, suggesting there may be a need to also reconsider the minimum LA recommendations for infants (Codex guideline currently set at 300 mg LA/100 kcal ≈ 6% of FA ≈ 2.7% EN; see Section 6) [65]. Furthermore, infants fed 0.55% EN from LA in the presence of ALA did not show any clinical signs of deficiency, according to a recent clinical study [61]. Rates of growth in length and weight measured during the first 3 months of life were identical to those of 20 exclusively breastfed (BF) infants. It is, however, of note that in BM, the LA content is much higher than 1% EN (Figure 1), so minimum requirements of LA are usually met when infants are BF.

The maximum level for LA intake, however, is more difficult to determine. A 4-to-6-months intervention study in infants fed formula with 45% of FAs as LA reported no obvious adverse health effects, but the study did report a two- to three-fold higher LA content in red blood cell membranes compared to BF infants (Table 1) [63]. Another study with an IF providing up to 58% of FAs as LA for 1 year resulted in very high LA levels in infant body fat (~45% FA), but again with no adverse effects [62]. Moreover, none of the adverse effects observed in preclinical or cohort studies (such as allergies, eczema, weight gain or neurocognitive outcomes) were reported in either of these studies [62].

In 1989, a UK expert group recognized that no adverse effects were observed in infants raised on a formula with almost 60% of FAs as LA, provided that the enhanced requirements for the anti-oxidant vitamin E were met [66]. However, the interventions in these studies lasted less than a year. Therefore, the expert group decided to play it safe, recommending that less than 25% of the total FAs (i.e., 1200 mg LA/100 kcal ≈ 24% of FA ≈ 10.8% EN) should consist of LA, forming the basis for the current recommendation by the European Union (Section 6). In China and by Codex, the boundary is set slightly higher: 1400 mg LA/100 kcal ≈ 28% FA ≈ 12.6% EN).

#### 5.3.2. LA/ALA Interventions to Study the Effect on Growth and Tolerance

Growth and tolerance were assessed in a number of different studies using LA/ALA ratios ranging from 4.8:1 to 44:1 in the absence of DHA (Table 2) [17,67,68,69,70]. IF containing canola oil with an LA/ALA ratio of 13:1 supported normal infant growth, as measured by weight and length gain compared to IF with an LA/ALA ratio of 5:1 (Table 2) [67]. Similar results were obtained by Makrides et al. testing formulas with ratios of 10:1 and 5:1 and by Ponder et al. testing formulas with ratios of 7:1 and 39:1 [17,67,69]. However, a clinical trial published by Jensen et al., showed that infants who received IF with an LA/ALA ratio of 4.8:1 (3.2% FA ALA) had a lower mean body weight at 4 months of age compared to infants who received IF with an LA/ALA ratio of 44:1 (0.4% FA ALA) [70], indicating that LA/ALA ratios lower than 5:1 should not be adopted until the effect of such ratios on growth are assessed more completely. In contrast, the higher ratios (up to 44:1) did not raise any concern regarding their effect on growth.

**Table 2:** LA/ALA intervention studies to determine the effect on infant growth and tolerance.

Author [Reference]	Tested Products	Study Population	Main Findings
Makrides et al. [17]	IFs with an LA/ALA ratio of either 10:1 (16.9%:1.7%) or 5:1 (16.3%:3.3%) vs. BF reference	Term infants (*n* = 36–37/group), from near birth to 34 wks of age	Lowering the LA/ALA in IF from 10:1 to 5:1 resulted in a modest increase in plasma DHA but had no effect on visual evoked potential acuity or growth rate. Tolerance was the same for both formulas.
Rzehak et al. [67]	Partially [pHF-W] or extensively hydrolyzed-whey [eHF-W] vs. regular cow’s milk IF [CMF]. LA/ALA ratios: pHF-W (13:1), eHF-W (5:1) and CMF (5:1).	Infants from first wk after birth to ≥120 days of age (pHF-W: *n* = 35; eHF-W: *n* = 32; CMF: *n* = 49)	All tested IFs with an LA/ALA ratio ranging between 5:1 and 13:1 supported normal infant growth as assessed by weight and length gain.
Jensen et al. [70]	4 IFs with LA (16% FA) and varying ALA concentrations (0.4%, 1.0%, 1.7%, or 3.2% FA) and LA/ALA ratios of 44:1, 18.2:1, 9.7:1 and 4.8:1	Term infants from birth until 240 days of age	The lowest LA/ALA ratio (or highest ALA level) resulted in higher plasma phospholipid DHA but was not associated with improved visual function. Mean body weight of infants who received the lowest LA/ALA ratio was less at 120 days, suggesting that LA/ALA ratios < 4.8 should not be adopted.
Ponder et al. [69]	Soybean (SOY) and corn (CORN) oil IF with an LA/ALA ratio of 7:1 (31.5%:4.8%) or 39:1 (34.2%:0.8%), respectively	Infants from first wk after birth to 8 wks of age (in total *n* = 43; SOY: *n* = 11; CORN: *n* = 14; HM: *n* = 18)	Growth did not differ between groups. Plasma phospholipid and RBC phosphatidylethanolamine DHA was similar in the CORN and SOY formula groups at all ages. The formula content of LA or the LA/ALA ratio had no effect on RBC or plasma DHA levels of the infants.

#### 5.3.3. LA/ALA Interventions that Attempt to Match the DHA Status in BF Infants

Some studies investigating IF with various combinations of ALA and LA have been carried out with the aim of mimicking the DHA status seen in BF infants. Although DHA levels in the majority of studies were marginally higher in infants fed formulas with a low LA/ALA ratio compared to infants fed higher ratios, they did not reach the values observed in BF infants; ARA levels might even be suppressed when low LA/ALA ratios are given (Table 2 and Table 3) [17,69,71,72,73]. These results confirm that adding DHA to formulas is more effective than increasing ALA content when it comes to maintaining red blood cell (RBC) DHA content. With the mandatory addition of DHA in current IFs, the contribution of ALA as a precursor to DHA is likely to be limited since dietary DHA itself consistently raises plasma DHA levels [11].

Only a few studies have tried to optimize DHA status via complementary feeding (Table 3). The introduction of complementary food usually leads to decreased intake of *n*-3 LCPUFAs, compared to exclusive breastfeeding. In order to test if DHA levels could be maintained, Libuda et al. tested the effects of complementary foods with different contents of ALA and DHA on term infant LCPUFA status. Healthy infants received complementary feeding twice weekly from the age of 4 to 6 months until the age of 10 months either with ALA-rich rapeseed (canola) oil, with salmon to provide DHA or with LA-rich corn oil (control). Regular salmon intake during complementary feeding improved infant EPA and DHA status. The use of rapeseed oil increased endogenous EPA synthesis but did not affect DHA status, while the LA-rich corn oil group (control) had the lowest LCPUFA status [74].

Another study used commercial complementary foods with distinct ratios of LA/ALA (10.7 vs. 14.8) to complement BM during the intervention period from 4 to 10 months. At the end of the intervention, the plasma concentrations of total *n*-3 FAs and of *n*-3 LCPUFAs, but not of ALA, were higher and the ratio of *n*-6/*n*-3 FAs was lower in the intervention group with the lower LA/ALA ratio [75]. Thus, intervention with ALA favored *n*-3 LCPUFA synthesis during complementary feeding when LCPUFA intake from BM and formula was decreasing.

**Table 3:** LA/ALA intervention studies that attempt to mimic the DHA status in breastfed infants.

Author [Reference]	Tested Products	Study Population	Main Findings
Jensen et al. [72]	4 IFs with LA (16% FA) and varying ALA concentrations (0.4%, 0.95%, 1.7%, or 3.2% FA) and LA/ALA ratios of 44:1, 18.2:1, 9.7:1 and 4.8:1)	Healthy term infants from shortly after birth until 120 days of age	Even though total n-3 FAs in plasma increased with higher levels of ALA, dietary LA/ALA ratios between 5 and 44 did not result in plasma levels of DHA similar to those at birth or in BF infants.
Chirouzo et al. [71]	First study: Two IFs with different FA compositions but no LCPUFAs. Second study: IF with or without DHA	Low-birth-weight infants after birth until the first 3 months of age	Both groups of IF-fed infants had significantly lower levels of DHA in RBCs compared with BF infants. In the second study, DHA remained stable in RBCs of infants supplemented with DHA, whereas it decreased in unsupplemented infants. Thus, adding DHA to IF is more effective in maintaining DHA levels in blood than increasing ALA content of IF.
Clark et al. [73]	Formula A: LA = 14%; ALA = 0.7%, Formula B: LA = 13%; ALA = 3.3% Formula C: LA= 3.5%; ALA = 1.1%	Healthy term infants from after birth until 10 weeks of age	Although DHA levels were higher in infants fed formula B and C with a low LA/ALA ratio compared to infants fed formula A with a high ratio, they did not reach the values observed in BF infants.
Libuda et al. [74]	Complementary foods with ALA-rich rapeseed oil, with DHA-rich salmon or with corn oil (control)	Healthy term infants from the age of 4 to 6 months until age of 10 months	Regular salmon consumption during complementary feeding enhanced infant EPA and DHA status, whereas use of rapeseed oil enhanced endogenous EPA synthesis, but didn’t affect DHA status compared to control.
Schwartz et al. [75]	Complementary meals with rapeseed oil (1.6 g/meal) rich in ALA (21% from study food)	Healthy term infants from 4 to 10 months. The control group (*n* = 53); the test group (*n* = 49).	After intervention, the plasma total *n*-3 FAs and *n*-3 LCPUFAs, but not ALA, were higher and the ratios of *n*-6/*n*-3 Fas were lower in the test group. Intervention with ALA favored *n*-3 LCPUFA synthesis in the complementary feeding period when LCPUFA intake from BM and formula was decreasing.
Sauerwald et al. [76]	Ifs with LA (16%), ALA (0.4%) and ARA (0.1%) but different DHA contents (from 0.04% to 0.52%) vs. BF with 11% LA, 0.1% ALA, 0.38% DHA	Preterms (*n* = 42, birth weight 1000–2200 g) From birth until day 28.	DHA supply increased plasma DHA in dose-dependent manner. IF DHA levels of 0.33% matched plasma DHA status of infants fed BM. LCPUFA synthesis was lower in BF infants than infants fed IF with different DHA and low ALA contents. With the LCPUFA supplementation used, DHA in formulas did not inhibit ARA or DHA synthesis.

In preterm infants, DHA supplied over a period of 28 days enhanced plasma DHA in a dose-dependent manner (Table 3) [76]. Plasma DHA status of infants fed IF with DHA levels of 0.33% FA matched the plasma DHA status of BF infants. LCPUFA synthesis was lower in BF infants than in those provided formulas with different DHA and low ALA contents (0.1%). DHA contents ranging from 0.04% to 0.52% in formulas did not inhibit ARA or DHA synthesis when LCPUFA supplementation was used (ALA = 0.4% and ARA= 0.1%) (Table 3).

#### 5.3.4. Cohort and LA/ALA Intervention Studies and Infant Health

An epidemiological study highlighted that maternal dietary *n*-6/*n*-3 PUFAs and LA/ALA intake during pregnancy and lactation were strongly correlated with the mental and psychomotor development in infants at 6 months of age (Table 4) [77]. Infants whose mothers’ BM had an LA/ALA ratio higher than 12 were roughly 1.5 to 2 times more likely to have a delayed performance on the mental and psychomotor development index compared to infants whose mothers’ BM had an LA/ALA ratio of less than 7. Thus, maintaining low ratios of *n*-6/*n*-3 PUFAs and of LA/ALA is encouraged for pregnant women. In contrast, maternal consumption of total *n*-6 and *n*-3 PUFAs, LA and ALA were not associated with these development parameters. Based on this epidemiological study, ratios seem to be more important than absolute values of LA and ALA.

Another large mother–child cohort study aimed to investigate the relationship between PUFA and LCPUFA levels in colostrum and cognition in early childhood (Table 4) [78]. A total of 709 BF children with available data on PUFA composition of BM were evaluated using parent-reported questionnaires for motor and language at 2 years of age, or global cognition at 3 years of age. High LA levels in colostrum (9.7–15.9% FA) were negatively associated with motor and cognitive scores at the age of 2 to 3 years, independent of BF duration. Children receiving BM with the highest levels of LA had a tendency to score closer to the never BF children than children BF with the lowest levels of LA (5.4–9.7% FA). No association was revealed between ARA and DHA in colostrum and child motor skills and cognition [78]. In contrast, a recent cohort study showed that maternal *n*-6 PUFAs were not related to child brain morphology, whereas exposure to lower *n*-3 LCPUFAs during fetal development was associated with smaller brain volume in childhood. This suggested that high LA levels may not have any negative impact on brain health, and that sufficient maternal *n*-3 LCPUFAs during pregnancy may be related to more optimal child brain development in the long term [79].

**Table 4:** Cohort and LA/ALA intervention studies and infant health.

Author [Reference]	Tested Products	Study Population	Main Findings
Kim et al. [77]	LA/ALA were 9.7% FA ± 6.3 and 11.1% FA ± 6.9	Pregnant women with a pre-pregnancy average BMI of 21.3 kg/m^2^	Both the maternal dietary *n*-6/*n*-3 PUFAs and LA/ALA intake were strongly correlated with the mental (MDI) and psychomotor development (PDI) of infants at 6 months of age. Thus, maintaining low *n*-6/*n*-3 PUFAs and LA/ALA is encouraged for women during pregnancy.
Bernard et al. [78]	Never BF versus 5.4–9.7% and 9.7–15.9% of LA in colostrum	BF children	LA levels were negatively associated with motor and cognitive scores, independent of BF duration. Children BF with the highest levels of LA tended to score closer to the never BF children than children BF with the lowest levels of LA.
Auestad et al. [80]	Formulas with LA (20% FA) and ALA (2% FA) with or without ARA + DHA (ARA 0.46% and DHA 0.14% FA)	Infants after birth until 1 year of age	The findings did not support adding ARA + DHA to IF to enhance growth, visual acuity, information processing, general development, language, or temperament in healthy term infants during the first 14 months after birth.

In contrast to epidemiological studies, intervention studies show less obvious results for the effects of PUFAs on cognition. Makrides et al. showed that lowering the LA/ALA ratio in formula from 10:1 to 5:1 by using low erucic acid canola oil (high in ALA) resulted in a modest rise in plasma DHA, but had no effect on visual acuity or growth rate (Table 2) [17]. Similarly, Jensen et al. showed that the lowest LA/ALA ratio (4.8 vs. 44) resulted in higher plasma DHA content at 4 months of age, but was not associated with improved visual function at 4 or 8 months of age. Moreover, mean body weight of infants who received the highest versus lowest ALA intake was lower at 4 months of age (Table 2) [70]. Lastly, evidence has been published suggesting that formulas containing a minimum of 1.75% FA as ALA and a ratio of LA/ALA of 5:1 to 15:1 may adequately support visual and cognitive development, despite lower and varying levels of ARA and DHA. For example, Auestad et al. showed that adding ARA and DHA to formulas containing 20% FA as LA and 2% FA as ALA was not necessary to enhance growth, visual acuity, information processing, general development, language or temperament in healthy term infants during the first 14 months after birth (Table 4) [80]. 

Although Auestad et al. indicated that the addition of DHA to formulas may not be required, during the last decades it has become clear that an adequate nutritional supply of DHA has direct health benefits for infants [81]. As a result, the most recent European and Chinese guidelines recommend the mandatory addition of DHA to IFs, which was optional in previous versions of these guidelines (see next Section). Furthermore, it is of note that with the mandatory presence of DHA in current IFs, the contribution of ALA as a precursor to DHA is likely to be restricted, since dietary DHA consistently raises plasma DHA levels. Unfortunately, no studies were found that investigated the effects of different ratios of LA/ALA or LA content in IF on infants in China.

## 6. Regulatory Context

The Codex Alimentarius international food standards of the Joint FAO/WHO Standards Programme (CXS 72-1981 and CXS 156-1987) are partly based on data on the FA composition of BM from healthy women, as well as observations from infant feeding studies. The Codex represents guidelines on adequate infant nutrition that are meant to provide guidance for national and regional food standards. The Codex guidelines are implemented in derived legislation in nearly all countries to ensure that IF is safe and meets the nutrient and energy requirements of developing infants. In addition, several national or regional authorities such as the U.S. Food and Drug Administration (FDA), the European Food Safety Authority (EFSA), Food Standards Australia New Zealand (FSANZ) and the Standardization Administration of China (SAC) provide guidance on IF compositions based on their own expert consultations.

In Table 5, the recommended levels for fat, LA, ALA, DHA and ARA are presented according to the latest Chinese (GB 10765–2021, GB 10766–2021 and GB 10767–2021), European (EC directive 2016/127) and Codex Alimentarius guidelines of the FAO/WHO (CXS 72–1981 and CXS 156-1987) [18,19]. For the recommended levels of LA, the EU maintains a narrower range compared to China and Codex. The range of LA concentrations in Stage 1 and 2 formulas is based on the level of LA intake (4% EN), which the EFSA panel had considered to be adequate for the majority of infants and the highest concentrations of LA observed in BM (24% FA) [60]. This altogether translates into a lower limit of 500 mg/100 kcal (equivalent to 4.5% EN) and an upper limit of 1200 mg/100 kcal (equivalent to 24% FA ≈10.8% EN).

The lowest level of ALA is similar for all three guidelines (Table 5). In line with the approach for LA, a lower limit for ALA in Stage 1 and 2 formulas can be derived based on the level of ALA intake (0.5% EN), which the EFSA panel had considered to be adequate for the majority of infants, and an upper limit can be derived based on the highest ALA levels observed in BM (2% FA) [63]. This altogether translates into a lower limit of 50 mg/100 kcal (equivalent to 0.5% EN) and an upper limit of 100 mg/100 kcal (equivalent to 0.9% EN). In the most recent European Commission (EC) directive (2016/127), there is not a specific ratio set for LA/ALA in the presence of LCPUFAs in Stage 1 and 2 formulas, while in the Chinese and Codex guidelines, the LA/ALA ratio in the formula is set to be between 5 and 15, with no maximum level set for ALA.

For DHA and ARA, the recommendations vary between the different stages as well as between regions. It is of note that no recommendations are provided in the EU and Codex guidelines for young child formulas (YCF = Stage 3 formula for children 12–36 months old) as experts do not believe that YCFs are needed [78], whereas China does have clear recommendations for YCFs (Stage 3). In China and other Asian countries, YCFs are quite popular.

In Table 6 and Table 7, some examples of registered formulas (before 2021) that are currently on the market in China are shown. These formula recipes all fall within the regulations. The formulas reported in Table 6 all contain LA concentrations that are at the lower end of the permitted range (376–794 mg/100 kcal), corresponding to 9–16% FA, whereas the average LA concentrations in BM in China are much higher (ranging between 22.6% and 21.4% FA depending on the region in China) [20]. The formulas reported in Table 7 contain higher levels of LA (17–22% FA), which are closer to the LA level in BM of Chinese mothers.

In 2021, a comprehensive review reported all of the available data on dietary intake of total fat, SFAs and individual PUFAs in children aged 1–7 years in different countries globally, and compared these values with recent FAO/WHO dietary recommended intake and EFSA dietary reference values [82]. The 65 studies were conducted in 33 countries worldwide and showed that total fat intake was generally lower than recommended in 1–3-year-old children (88% of studies) and total SFA intake was higher than the limit of 10% EN in about 70% of children aged 2–7 years. Strikingly, LA intake was below FAO/WHO recommendations in 24% of studies in younger children (1–2 years) but was within the range of FAO/WHO recommendations for older children (2–7 years). However, LA intake was lower than EFSA recommendations in about half of the studies for all age groups (1–7 years), including in rural China [82]. ALA intake was below FAO/WHO and EFSA recommendations in almost half of the studies for all age groups, including in rural China. DHA (or EPA + DHA) intake was lower than FAO/WHO recommendations in most studies (especially for ages 1–2 years and 5–7 years), and lower than EFSA recommendations in all studies of children more than 2 years old [82].

## 7. Gaps in Knowledge

Currently, there are no toxicity data regarding LA [60]. This is why authorities such as EFSA, FAO/WHO and SAC cannot set an upper safe level of intake for LA. 

A clear gap in knowledge exists regarding the possible impact of LA and ALA levels in IF in the presence of LCPUFAs (DHA and ARA), as in the current formulas that are on the market (e.g., in China and the EU).

An urgent need exists for well-designed clinical intervention trials to create clarity about optimal and safe levels of LA in the context of the current IF market and to determine long-term implications on functional health outcomes. 

In particular, there is a need for dose-response intervention studies (including LA concentrations currently found in Chinese BM (e.g., LA 22% of FA) but staying within the current regulatory boundaries) in order to determine if, and at what level, LA induces potential adverse effects and/or has beneficial effects. 

The aforementioned gaps are the same whether looked at from an Eastern or Western perspective.

## 8. Discussion

The requirements for LA in IF may best be described as a range rather than a single value, due to the individual variability in FA status at birth. Generally, it is assumed that the estimated average requirement ± 2 standard deviations of the variation in infant requirements will cover the needs of almost all healthy individuals in a population. Crossing either side of the lower or upper margin could increase the risk of negative consequences for the infant’s metabolism, physiological functions and short- and long-term health outcomes [11]. However, the current evidence is too limited to provide a strong rationale for clear cutoff values for LA. Therefore, the current recommendations are range estimates and are based on the limited available evidence including from intervention and cohort studies in infants and from concentrations currently found in BM.

As described in Section 5, the role of LA in fetal and infant growth is of major importance. LA is an essential fatty acid, as it cannot be synthesized by the body. It is required to maintain “metabolic integrity” [60]. LA plays a key role as the precursor of ARA, which is essential for normal growth and development of the brain. In term infants, an inadequate concentration of LA has been shown to lead to suboptimal growth and/or skin health issues.

On the basis of the studies mentioned in Table 1, the minimum requirement for LA of 1% of EN in IF was commonly accepted by authorities such as the FAO/WHO and extended to 2.7% of EN (=300 mg/100 kcal) to ensure that the dietary requirement of infants is met. This forms the basis of the Codex [18,19] and Chinese guidelines. For the EU, the requirements are set higher (4.5% of EN = 500 mg/100 kcal), which is in line with the recommendations made by an International Expert Group coordinated by The European Society for Pediatric Gastroenterology, Hepatology and Nutrition (ESPGHAN) [83,84].

Lowering of the minimum requirements of LA might be considered because a clinical study in infants fed IF with LA at 0.55% of EN in the presence of ALA did not show any clinical signs of deficiency (Table 1) [61]. However, only that single study indicated a lack of any adverse effects at this low dose [61]. An LA level of 0.1% of EN did show negative effects on skin health, but the effects could be reversed by adding LA at 1% of EN [57,58,59]. However, it is of note that the LA content in BM is much higher than 1% of EN (Figure 1, the lowest average LA found in BM is 8.5% of FA ≈3.8% EN), so minimum requirements for LA are usually met when infants are BF. In China, the lowest average LA level found in BM was 10% of FA ≈ 4.5% EN [85]. Furthermore, in rural China, the mean intake of LA among children aged 1–7 years was ≈3% EN according to a recent systematic review [82]. Thus, reducing the minimum requirement of LA might not be necessary, and the current recommendations for the minimum requirements are adequate for Codex countries and China (300 mg LA/100 kcal ≈ 6% FA ≈ 2.7% EN) and for the EU (500 mg LA/100 kcal ≈ 10% FA ≈ 4.5% of EN).

What the upper level of LA intake should be is unclear due to the absence of relevant toxicity data and the absence of clinical data regarding the potential impact of LA and ALA levels in IF in the presence of LCPUFAs (DHA and ARA) as in the current formulas that are on the market (e.g., in China and the EU).

The current maximum LA levels are set on the basis of the available studies (Table 1 and Table 2) [62,63,69] and the opinion of a UK expert group [66]. A recent international expert group did not find any scientific reason to increase the maximum permitted LA level [11]. On the contrary, they concluded that the available preclinical information suggests potential disadvantages of high LA intake in the early postnatal period [11]. According to these experts, the current maximum value set for LA content is considered necessary, because high intakes may induce untoward metabolic effects with respect to lipoprotein metabolism, immune function, eicosanoid balance, and oxidative stress [11].

The current highest maximum set by authorities (e.g., Codex or China) is 1400 mg LA/100 kcal ≈ 28% FA ≈ 12.6% EN. There is no need to go beyond this maximum according to the current scientific evidence. Firstly, no toxicological data are available highlighting the No Adverse Effect Level (NOAEL). Secondly, there is no definitive proof of long-term safety for going beyond this maximum. Virtually all intervention studies in infants lasted less than 1.5 years, and most of them were done in the absence of added DHA and ARA, which is not representative of the current IF on the market in China, the EU and many other countries. Thirdly, the current average BM LA level in most countries (Figure 1)—including China (~22% FA), which is high compared to the rest of the world—falls well within the current recommended range.

Regarding the ratio of LA/ALA, it is clear that going below a ratio of 5:1 is not advisable because of safety concerns. Such low ratios may affect growth in infants [70]. In contrast, higher ratios (up to LA/ALA 44:1) did not raise any concerns regarding their effect on growth (Table 2) [70]. In the absence of added DHA to formulas, ratios closer to 5:1 than to 15:1 are preferable, as they more effectively promote the endogenous synthesis of DHA. However, the DHA levels in IF-fed infants never reach values observed in BF infants, and no effect on vision was reported [17,70]. Therefore, it is now mandatory to add DHA to IF in China and the EU to maintain DHA levels comparable to BM.

Some examples of IFs currently on the market in China (Table 7) contain high levels of LA (17–22% FA) which are closer to the LA level in BM of Chinese mothers (mean LA = 22% FA). These IFs have an LA/ALA ratio of around 10:1 (Table 6 and Table 7).

Lastly, the influence of the LA and LA/ALA ratio in IF on health in early life cannot be adequately studied independent of the levels of DHA and other *n*-3 PUFAs, as it is about the balance between these compounds. Unfortunately, most studies have been carried out in the absence of any added DHA, which is not representative of the IFs currently on the market.

## 9. Conclusions

There is a clear gap in knowledge regarding the potential impact of LA and ALA levels in IF in the presence of LCPUFAs (DHA and ARA), as in the current formulas that are on the market (e.g., in China and the EU). Even though the science is limited and no longer representative of the current IF market, there are no scientific reasons to go beyond the recommended ranges for LA and ALA provided by authorities such as Codex, the Chinese government and the European Commission, only reasons to stay within these ranges. In the EU and in China, it is mandatory to add DHA to IF. It is advisable to add DHA to IF in an amount that is as close to BM DHA level as possible because even low ratios of LA/ALA did not trigger sufficient endogenous DHA synthesis to match BF DHA levels. The health benefits of DHA are well recognized by the scientific community and well understood by clinicians and parents.

## Figures and Tables

**Table 5:** Recommended minimum and maximum levels for fat, LA, ALA, DHA and ARA according to the latest Chinese, European and Codex guidelines.

	China			EU		Codex	
Nutrient/100 kcal	Stage 1 0–6 mo	Stage 2 6–12 mo	Stage 3 12–36 mo	Stage 1 0–6 mo	Stage 2 6–12 mo	Stage 1 0–6 mo	Stage 2 6–12 mo
Fat, g	4.4–6.0	3.5–6.0	3.5–6.0	4.4–6.0	4.4–6.0	4.4–6.0	3.0–6.0
LA, g	0.3–1.4	0.3–1.4	0.3–1.4	0.5–1.2	0.5–1.2	0.3–1.4 ^1^	0.3–N.S.
ALA, mg	50–N.S.	50–N.S.	50–N.S.	50–100	50–100	50-N.S.	N.S.
LA/ALA	5:1–15:1	5:1–15:1	5:1–15:1	N.S.	N.S.	5:1–15:1	N.S.
DHA, mg	15–40	15–40	N.S.–40	20–50	20–50	N.S.–22 ^2^	N.S.
ARA, mg	N.S.–80	N.S.–80	N.S.–80	N.S.	N.S.	N.S.^2^	N.S.

^1^ Only a guidance upper level (GUL), not a strict maximum, is provided for LA. ^2^ Codex defines a GUL for DHA of 0.5% of total FAs (22 mg/100 kcal, 0.2 EN%) and specifies that the DHA content should be matched with at least equal levels of ARA. Mo.: Months, N.S.: not specified.

**Table 6:** FA compositions of some infant and young child formulas on the Chinese market.

	Company A ^1^			Company B ^2^			Company C ^3^		
Nutrient	Stage 1 0–6 mo	Stage 2 6–12 mo	Stage 3 12–36 mo	Stage 1 0–6 mo	Stage 2 6–12 mo	Stage 3 12–36 mo	Stage 1 0–6 mo	Stage 2 6–12 mo	Stage 3 12–36 mo
Fat, g/100 kcal	5.1	4.6	4.2	5.4	4.5	3.7	5.4	4.6	4.2
LA, mg/100 kcal	543	418	376	794	627	711	794	543	418
ALA, mg/100 kcal	59	NL	NL	64	NL	NL	79	NL	NL
LA/ALA	9.2			12.4			10.1		
DHA % FA	0.22	0.18	0.05	0.36	0.4	0.34	0.17	0.12	0.15
ARA % FA	0.28	0.24	0.11	0.36	0.4	0.09	0.35	0.15	0.30

^1^ Company A: Junlebao (Lezhen); ^2^ Company B: Wyeth (Ultima); ^3^ Company C: Firmus (Xingfeifan). Mo.: Months, NL: none on the label.

**Table 7:** FA compositions of some infant and young child formulas on the Chinese market.

	Company Y ^4^			Company Z ^5^		
Nutrient	Stage 1 0–6 mo	Stage 2 6–12 mo	Stage 3 12–36 mo	Stage 1 0–6 mo	Stage 2 6–12 mo	Stage 3 12–36 mo
Fat, g/100 kcal	5.4	4.6	4.6	5.2	4.8	3.7
LA, mg/100 kcal	920	586	586	1046	920	711
ALA, mg/100 kcal	90	NL	NL	110	NL	NL
LA/ALA	10	-	-	10	-	-
DHA % FA	0.32	0.30	0.32	0.21	0.18	0.21
ARA % FA	0.64	0.60	0.64	0.38	0.33	0.38

^4^ Company Y: Beingmate (Aijia); ^5^ Company Z: Ausnutria (Hyproca 1897); Mo.: Months, NL: none on the label.

## Supplementary Materials

The following supporting information can be downloaded at: https://www.mdpi.com/article/10.3390/nu15092187/s1, Table S1: Average PUFA levels (% of total fatty acids) and the ratio of LA to ALA in breast milk reported in studies from different countries. References [20,31,41,86,87,88,89,90,91,92,93,94,95,96,97,98,99,100,101,102,103,104,105,106,107,108,109,110,111,112,113,114,115,116,117,118,119,120,121,122,123,124,125,126,127,128] are cited in the supplementary materials.

## Abbreviations

α-linolenic acid (ALA), arachidonic acid (ARA), breast milk (BM), docosahexaenoic acid (DHA), the European Food Safety Authority (EFSA), energy (EN), fatty acid (FA), Food and Drug Administration (FDA), Food Standards Australia New Zealand (FSANZ), infant formula (IF), long-chain polyunsaturated fatty acids (LCPUFAs), linoleic acid (LA), 1,3-dioleoyl-2-palmitoylglycerol (OPO), 1-oleoyl-2-palmitoyl-3-linoleoylglycerol (OPL), polyunsaturated fatty acids (PUFAs), and the Standardization Administration of China (SAC)

## References

[1] Ballard O., Morrow A.L. (2013). Human Milk Composition. Nutrients and Bioactive Factors. Pediatr. Clin. N. Am..

[2] Martin C.R., Ling P.-R., Blackburn G.L. (2016). Review of Infant Feeding: Key Features of Breast Milk and Infant Formula. Nutrients.

[3] Turck D., Bresson J., Burlingame B., Dean T., Fairweather-Tait S., Heinonen M., Hirsch-Ernst K.I., Mangelsdorf I., McArdle H.J., Naska A. (2017). Scientific Opinion on the Safety and Suitability for Use by Infants of Follow-on Formulae with a Protein Content of at Least 1.6 g/100 Kcal. EFSA J..

[4] Patro-Golab B., Zalewski B.M., Kouwenhoven S.M., Karas J., Koletzko B., Bernard van Goudoever J., Szajewska H. (2016). Protein Concentration in Milk Formula, Growth, and Later Risk of Obesity: A Systematic Review. J. Nutr..

[5] Bode L. (2012). Human Milk Oligosaccharides: Every Baby Needs a Sugar Mama. Glycobiology.

[6] Koletzko B., Agostoni C., Bergmann R., Ritzenthaler K., Shamir R. (2011). Physiological Aspects of Human Milk Lipids and Implications for Infant Feeding: A Workshop Report. Acta Paediatr..

[7] O’Brien J.S., Sampson E.L. (1965). Lipid Composition of the Normal Human Brain: Gray Matter, White Matter, and Myelin. J. Lipid Res..

[8] Benatti P., Nicolai R., Calvani M., Peluso G. (2004). Polyunsaturated Fatty Acids: Biochemical, Nutritional and Epigenetic Properties. J. Am. Coll. Nutr..

[9] Martínez M., Mougan I. (1998). Fatty Acid Composition of Human Brain Phospholipids during Normal Development. J. Neurochem..

[10] Floris L.M., Stahl B., Abrahamse-Berkeveld M., Teller I.C. (2020). Human Milk Fatty Acid Profile across Lactational Stages after Term and Preterm Delivery: A Pooled Data Analysis. Prostaglandins Leukot. Essent. Fatty Acids.

[11] Carlson S.E., Schipper L., Brenna J.T., Agostoni C., Calder P.C., Forsyth S., Legrand P., Abrahamse-Berkeveld M., van de Heijning B.J.M., van der Beek E.M. (2021). Perspective: Moving Toward Desirable Linoleic Acid Content in Infant Formula. Adv. Nutr..

[12] Ailhaud G., Massiera F., Weill P., Legrand P., Alessandri J.M., Guesnet P. (2006). Temporal Changes in Dietary Fats: Role of n-6 Polyunsaturated Fatty Acids in Excessive Adipose Tissue Development and Relationship to Obesity. Prog. Lipid Res..

[13] OECD-FAO Agricultural Outlook (Edition 2021)|OECD Agriculture Statistics|OECD ILibrary. https://www.oecd-ilibrary.org/agriculture-and-food/data/oecd-agriculture-statistics/oecd-fao-agricultural-outlook-edition-2021_4bde2d83-en?parentId=http%3A%2F%2Finstance.metastore.ingenta.com%2Fcontent%2Fcollection%2Fagr-data-en.

[14] Oléagineuse L.F., Asie E.N., Mittaine J.-F. (2016). Oilseeds and Vegetable Oils in Asia: A World of Diversity. OCL.

[15] Delplanque B., Gibson R., Koletzko B., Lapillonne A., Strandvik B. (2015). Lipid Quality in Infant Nutrition: Current Knowledge and Future Opportunities. J. Pediatr. Gastroenterol. Nutr..

[16] Delplanque B., Du Q., Martin J.C., Guesnet P. (2018). Lipids for Infant Formulas. OCL—Oilseeds Fats Crops Lipids.

[17] Makrides M., Neumann M.A., Jeffrey B., Lien E.L., Gibson R.A. (2000). A Randomized Trial of Different Ratios of Linoleic to α-Linolenic Acid in the Diet of Term Infants: Effects on Visual Function and Growth. Am. J. Clin. Nutr..

[18] (2011). Standard for Infant Formula and Formulas for Special Medicalpurposes Intended for Infants Sections A&B Revision 2007. Amended 2011.

[19] (2017). World Health Organisation (WHO) Standard for Follow-Up Formula.

[20] Sun H., Ren Q., Zhao X., Tian Y., Pan J., Wei Q., Li Y., Chen Y., Zhang H., Zhang W. (2020). Regional Similarities and Differences in Mature Human Milk Fatty Acids in Chinese Population: A Systematic Review. Prostaglandins Leukot. Essent. Fat. Acids.

[21] Ren Q., Zhou Y., Zhang W., Tian T., Sun H., Zhao X., Xu Y., Jiang S. (2021). Longitudinal changes in the bioactive proteins in human milk of the Chinese population: A systematic review. Food Sci. Nutr..

[22] Peng Y., Zhou T., Wang Q., Liu P., Zhang T., Zetterström R., Strandvik B. (2009). Fatty Acid Composition of Diet, Cord Blood and Breast Milk in Chinese Mothers with Different Dietary Habits. Prostaglandins Leukot. Essent. Fat. Acids.

[23] Stark A.H., Crawford M.A., Reifen R. (2008). Update on Alpha-Linolenic Acid. Nutr. Rev..

[24] Miles E.A., Calder P.C. (2017). The Influence of the Position of Palmitate in Infant Formula Triacylglycerols on Health Outcomes. Nutr. Res..

[25] Tu A., Ma Q., Bai H., Du Z. (2017). A Comparative Study of Triacylglycerol Composition in Chinese Human Milk within Different Lactation Stages and Imported Infant Formula by SFC Coupled with Q-TOF-MS. Food Chem..

[26] Sun C., Wei W., Su H., Zou X., Wang X. (2018). Evaluation of Sn-2 Fatty Acid Composition in Commercial Infant Formulas on the Chinese Market: A Comparative Study Based on Fat Source and Stage. Food Chem..

[27] Kallio H., Nylund M., Boström P., Yang B. (2017). Triacylglycerol Regioisomers in Human Milk Resolved with an Algorithmic Novel Electrospray Ionization Tandem Mass Spectrometry Method. Food Chem..

[28] Yuan T., Qi C., Dai X., Xia Y., Sun C., Sun J., Yu R., Zhou Q., Jin Q., Wei W. (2019). Triacylglycerol Composition of Breast Milk during Different Lactation Stages. J. Agric. Food Chem..

[29] Wu W., Balter A., Vodsky V., Odetallh Y., Ben-Dror G., Zhang Y., Zhao A. (2021). Chinese Breast Milk Fat Composition and Its Associated Dietary Factors: A Pilot Study on Lactating Mothers in Beijing. Front. Nutr..

[30] Giuffrida F., Cruz-Hernandez C., Bertschy E., Fontannaz P., Elmelegy I.M., Tavazzi I., Marmet C., Sanchez-Bridge B., Thakkar S.K., de Castro C.A. (2016). Temporal Changes of Human Breast Milk Lipids of Chinese Mothers. Nutrients.

[31] Martin M.A., Lassek W.D., Gaulin S.J.C., Evans R.W., Woo J.G., Geraghty S.R., Davidson B.S., Morrow A.L., Kaplan H.S., Gurven M.D. (2012). Fatty Acid Composition in the Mature Milk of Bolivian Forager-Horticulturalists: Controlled Comparisons with a US Sample. Matern. Child. Nutr..

[32] Szabó É., Boehm G., Beermann C., Weyermann M., Brenner H., Rothenbacher D., Decsi T. (2010). Fatty Acid Profile Comparisons in Human Milk Sampled from the Same Mothers at the Sixth Week and the Sixth Month of Lactation. J. Pediatr. Gastroenterol. Nutr..

[33] Havlicekova Z., Jesenak M., Banovcin P., Kuchta M. (2016). Beta-Palmitate—A Natural Component of Human Milk in Supplemental Milk Formulas. Nutr. J..

[34] Zou L., Pande G., Akoh C.C. (2016). Infant Formula Fat Analogs and Human Milk Fat: New Focus on Infant Developmental Needs. Annu. Rev. Food Sci. Technol..

[35] Bar-Yoseph F., Lifshitz Y., Cohen T. (2013). Review of Sn-2 Palmitate Oil Implications for Infant Health. Prostaglandins Leukot. Essent. Fat. Acids.

[36] Zhang N., Zeng J.P., Wu Y.P., Wei M., Zhang H., Zheng L., Deng Z.Y., Li J. (2021). Human Milk Sn-2 Palmitate Triglyceride Rich in Linoleic Acid Had Lower Digestibility but Higher Absorptivity Compared with the Sn-2 Palmitate Triglyceride Rich in Oleic Acid In Vitro. J. Agric. Food Chem..

[37] Sanders T.A.B., Reddy S. (1992). The Influence of a Vegetarian Diet on the Fatty Acid Composition of Human Milk and the Essential Fatty Acid Status of the Infant. J. Pediatr..

[38] Chulei R., Xiaofang L., Hongsheng M., Xiulan M., Guizheng L., Gianhong D., DeFrancesco C.A., Connor W.E. (1995). Milk Composition in Women from Five Different Regions of China: The Great Diversity of Milk Fatty Acids. J. Nutr..

[39] Kneebone G.M., Kneebone R., Gibson R.A. (1985). Fatty Acid Composition of Breast Milk from Three Racial Groups from Penang, Malaysia. Am. J. Clin. Nutr..

[40] Fang C., Beghin J.C. (2002). Urban Demand for Edible Oils and Fats in China: Evidence from Household Survey Data. J. Comp. Econ..

[41] Miliku K., Duan Q.L., Moraes T.J., Becker A.B., Mandhane P.J., Turvey S.E., Lefebvre D.L., Sears M.R., Subbarao P., Field C.J. (2019). Human Milk Fatty Acid Composition Is Associated with Dietary, Genetic, Sociodemographic, and Environmental Factors in the CHILD Cohort Study. Am. J. Clin. Nutr..

[42] Liu G., Ding Z., Li X., Chen X., Wu Y., Xie L. (2016). Relationship between Polyunsaturated Fatty Acid Levels in Maternal Diets and Human Milk in the First Month Post-Partum. J. Hum. Nutr. Diet..

[43] Liu Y., Liu X., Wang L., Perovic M. (2019). The Investigation of Fatty Acid Composition of Breast Milk and Its Relationship with Dietary Fatty Acid Intake in 5 Regions of China. Medicine.

[44] Tian H.M., Wu Y.X., Lin Y.Q., Chen X.Y., Yu M., Lu T., Xie L. (2019). Dietary Patterns Affect Maternal Macronutrient Intake Levels and the Fatty Acid Profile of Breast Milk in Lactating Chinese Mothers. Nutrition.

[45] Chen H., Wang P., Han Y., Ma J., Troy F.A., Wang B., Wang B. (2012). Evaluation of Dietary Intake of Lactating Women in China and Its Potential Impact on the Health of Mothers and Infants. BMC Womens Health.

[46] dos Santos Q., Sichieri R., Marchioni D.M., Verly Junior E. (2014). Brazilian Pregnant and Lactating Women Do Not Change Their Food Intake to Meet Nutritional Goals. BMC Pregnancy Childbirth.

[47] Sotres-Alvarez D., Herring A.H., Siega-Riz A.M. (2013). Latent Transition Models to Study Women’s Changing of Dietary Patterns from Pregnancy to 1 Year Postpartum. Am. J. Epidemiol..

[48] Cucó G., Fernández-Ballart J., Sala J., Viladrich C., Iranzo R., Vila J., Arija V. (2006). Dietary Patterns and Associated Lifestyles in Preconception, Pregnancy and Postpartum. Eur. J. Clin. Nutr..

[49] Crozier S.R., Robinson S.M., Godfrey K.M., Cooper C., Inskip H.M. (2009). Women’s Dietary Patterns Change Little from before to during Pregnancy. J. Nutr..

[50] Bobiński R., Bobińska J. (2020). Fatty Acids of Human Milk—A Review. Int. J. Vitam. Nutr. Res..

[51] Carnielli V.P., Wattimena D.J.L., Luijendijk I.H.T., Boerlage A., Degenhart H.J., Sauer P.J.J. (1996). The Very Low Birth Weight Premature Infant Is Capable of Synthesizing Arachidonic and Docosahexaenoic Acids from Linoleic and Linolenic Acids. Pediatr. Res..

[52] Sauerwald T.U., Hachey D.L., Jensen C.L., Chen H., Anderson R.E., Heird W.C. (1997). Intermediates in Endogenous Synthesis of C22:6ω3 and C20:4ω6 by Term and Preterm Infants. Pediatr. Res..

[53] Klevebro S., Juul S.E., Wood T.R. (2020). A More Comprehensive Approach to the Neuroprotective Potential of Long-Chain Polyunsaturated Fatty Acids in Preterm Infants Is Needed—Should We Consider Maternal Diet and the n-6:N-3 Fatty Acid Ratio?. Front. Pediatr..

[54] Su H., Liu R., Chang M., Huang J., Wang X. (2017). Dietary Linoleic Acid Intake and Blood Inflammatory Markers: A Systematic Review and Meta-Analysis of Randomized Controlled Trials. Food Funct..

[55] Johnson G.H., Fritsche K. (2012). Effect of Dietary Linoleic Acid on Markers of Inflammation in Healthy Persons: A Systematic Review of Randomized Controlled Trials. J. Acad. Nutr. Diet..

[56] Cole R.M., Puchala S., Ke J.Y., Abdel-Rasoul M., Harlow K., O’Donnell B., Bradley D., Andridge R., Borkowski K., Newman J.W. (2020). Linoleic Acid–Rich Oil Supplementation Increases Total and High-Molecular-Weight Adiponectin and Alters Plasma Oxylipins in Postmenopausal Women with Metabolic Syndrome. Curr. Dev. Nutr..

[57] Hansen A.E., Haggard M.E., Boelsche A.N., Adam D.J., Wiese H.F. (1958). Essential Fatty Acids in Infant Nutrition. III. Clinical Manifestations of Linoleic Acid Deficiency. J. Nutr..

[58] Wiese H.F., Hansen A.E., Adam D.J. (1958). Essential Fatty Acids in Infant Nutrition. I. Linoleic Acid Requirement in Terms of Serum Di-, Tri- and Tetraenoic Acid Levels. J. Nutr..

[59] Hansen E., Wiese F., Adam J.D. (1963). Role of Linoleic Acid in Infant Nutrition. Pediatrics.

[60] EFSA Panel on Dietetic Products, Nutrition, and Allergies (NDA) (2016). Scientific Opinion on Dietary Reference Values for Fats, Including Saturated Fatty Acids, Polyunsaturated Fatty Acids, Monounsaturated Fatty Acids, Trans Fatty Acids, and Cholesterol. EFSA J..

[61] Naismith D.J., Deeprose S.P., Supramaniam G., Williams M.J.H. (1978). Reappraisal of Linoleic Acid Requirement of the Young Infant, with Particular Regard to Use of Modified Cows’ Milk Formulae. Arch. Dis. Child..

[62] Widdowson E.M., Dauncey M.J., Gairdner D.M.T., Jonxis J.H.P., Pelikan-Filípková M. (1975). Body Fat of British and Dutch Infants. Br. Med. J..

[63] Putnam J.C., Carlson S.E., D.DeVoe P.W., Barness L.A. (1982). The Effect of Variations in Dietary Fatty Acids on the Fatty Acid Composition of Erythrocyte Phosphatidylcholine and Phosphatidylethanolamine in Human Infants. Am. J. Clin. Nutr..

[64] Choque B., Catheline D., Rioux V., Legrand P. (2014). Linoleic Acid: Between Doubts and Certainties. Biochimie.

[65] Choque B., Catheline D., Delplanque B., Guesnet P., Legrand P. (2015). Dietary Linoleic Acid Requirements in the Presence of α-Linolenic Acid Are Lower than the Historical 2% of Energy Intake Value, Study in Rats. Br. J. Nutr..

[66] Widdowson E.M. (1989). Upper Limits of Intakes of Total Fat and Polyunsaturated Fatty Acids in Infant Formulas1. J. Nutr..

[67] Rzehak P., Koletzko S., Koletzko B., Sausenthaler S., Reinhardt D., Grübl A., Bauer C.P., Krämer U., Bollrath C., von Berg A. (2011). Growth of Infants Fed Formula Rich in Canola Oil (Low Erucic Acid Rapeseed Oil). Clin. Nutr..

[68] van Egmond A.W.A., Kosorok M.R., Koscik R., Laxova A., Farrell P.M. (1996). Effect of Linoleic Acid Intake on Growth of Infants with Cystic Fibrosis. Am. J. Clin. Nutr..

[69] Ponder D.L., Innis S.M., Benson J.D., Siegman J.S. (1992). Docosahexaenoic Acid Status of Term Infants Fed Breast Milk or Infant Formula Containing Soy Oil or Corn Oil. Pediatr. Res..

[70] Jensen C.L., Prager T.C., Fraley J.K., Chen H., Anderson R.E., Heird W.C. (1997). Effect of Dietary Linoleic/Alpha-Linolenic Acid Ratio on Growth and Visual Function of Term Infants. J. Pediatr..

[71] Chirouze V., Lapillonne A., Putet G., Salle B. (1994). Red Blood Cell Fatty Acid Composition in Low-birth-weight Infants Fed Either Human Milk or Formula during the First Months of Life. Acta Paediatr..

[72] Jensen C.L., Chen H., Fraley J.K., Anderson R.E., Heird W.C. (1996). Biochemical Effects of Dietary Linoleic/α-Linolenic Acid Ratio in Term Infants. Lipids.

[73] Clark K.J., Makrides M., Neumann M.A., Gibson R.A. (1992). Determination of the Optimal Ratio of Linoleic Acid to α-Linolenic Acid in Infant Formulas. J. Pediatr..

[74] Libuda L., Mesch C.M., Stimming M., Demmelmair H., Koletzko B., Warschburger P., Blanke K., Reischl E., Kalhoff H., Kersting M. (2016). Fatty Acid Supply with Complementary Foods and LC-PUFA Status in Healthy Infants: Results of a Randomised Controlled Trial. Eur. J. Nutr..

[75] Schwartz J., Dube K., Sichert-Hellert W., Kannenberg F., Kunz C., Kalhoff H., Kersting M. (2009). Modification of Dietary Polyunsaturated Fatty Acids via Complementary Food Enhances N-3 Long-Chain Polyunsaturated Fatty Acid Synthesis in Healthy Infants: A Double Blinded Randomised Controlled Trial. Arch. Dis. Child..

[76] Sauerwald U., Fink M.M., Demmelmair H., Schoenaich P.V., Rauh-Pfeiffer A.A.M., Koletzko B. (2012). Effect of Different Levels of Docosahexaenoic Acid Supply on Fatty Acid Status and Linoleic and α-Linolenic Acid Conversion in Preterm Infants. J. Pediatr. Gastroenterol. Nutr..

[77] Kim H., Kim H., Lee E., Kim Y., Ha E.H., Chang N. (2017). Association between Maternal Intake of N-6 to n-3 Fatty Acid Ratio during Pregnancy and Infant Neurodevelopment at 6 Months of Age: Results of the MOCEH Cohort Study. Nutr. J..

[78] Bernard J.Y., Armand M., Garcia C., Forhan A., de Agostini M., Charles M.-A., Heude B. (2015). The Association between Linoleic Acid Levels in Colostrum and Child Cognition at 2 and 3 y in the EDEN Cohort. Pediatr. Res..

[79] Zou R., el Marroun H., Voortman T., Hillegers M., White T., Tiemeier H. (2021). Maternal Polyunsaturated Fatty Acids during Pregnancy and Offspring Brain Development in Childhood. Am. J. Clin. Nutr..

[80] Auestad N., Halter R., Hall R.T., Blatter M., Bogle M.L., Burks W., Erickson J.R., Fitzgerald K.M., Dobson V., Innis S.M. (2001). Growth and Development in Term Infants Fed Long-Chain Polyunsaturated Fatty Acids: A Double-Masked, Randomized, Parallel, Prospective, Multivariate Study. Pediatrics.

[81] Brenna J.T., Carlson S.E. (2014). Docosahexaenoic Acid and Human Brain Development: Evidence That Adietary Supply Is Needed for Optimal Development. J. Hum. Evol..

[82] Monnard C., Fleith M. (2021). Total Fat and Fatty Acid Intake among 1–7-Year-Old Children from 33 Countries: Comparison with International Recommendations. Nutrients.

[83] EFSA Panel on Dietetic Products Nutrition and Allergies (NDA) (2014). Scientific Opinion on the Essential Composition of Infant and Follow-on Formulae. EFSA J..

[84] Koletzko B., Baker S., Cleghorn G., Fagundes Neto U., Gopalan S., Hernell O., Seng Hock Q., Jirapinyo P., Lonnerdal B., Pencharz P. (2005). Global Standard for the Composition of Infant Formula: Recommendations of an ESPGHAN Coordinated International Expert Group Background of the Espghan Coordinated International Expert Group Consultation. J. Pediatr. Gastroenterol. Nutr..

[85] Xiang M., Alfvén G., Blennow M., Trygg M., Zetterström R. (2000). Long-Chain Polyunsaturated Fatty Acids in Human Milk and Brain Growth during Early Infancy. Acta Paediatr. Int. J. Paediatr..

[86] Mitoulas L.R., Gurrin L.C., Doherty D.A., Sherriff J.L., Hartmann P.E. (2003). Infant Intake of Fatty Acids from Human Milk over the First Year of Lactation. Br. J. Nutr..

[87] Berenhauser A.C., Pinheiro Do Prado A.C., da Silva R.C., Gioielli L.A., Block J.M. (2012). Fatty Acid Composition in Preterm and Term Breast Milk. Int. J. Food Sci. Nutr..

[88] Nishimura R.Y., de Castro G.S.F., Jordão A.A., Sartorelli D.S. (2013). Breast Milk Fatty Acid Composition of Women Living Far from the Coastal Area in Brazil. J. Pediatr. (Rio J.).

[89] Patin R.V., Vítolo M.R., Valverde M.A., Carvalho P.O., Pastore G.M., Ancona Lopez F. (2006). The Influence of Sardine Consumption on the Omega-3 Fatty Acid Content of Mature Human Milk. J. Pediatr..

[90] Innis S.M., King D.J. (1999). Trans Fatty Acids in Human Milk Are Inversely Associated with Concentrations of Essential All-Cis n-6 and n-3 Fatty Acids and Determine Trans, but Not n-6 and n-3, Fatty Acids in Plasma Lipids of Breast-Fed Infants. Am. J. Clin. Nutr..

[91] Tijerina-Sáenz S., Innis S.M., Kitts D.D. (2009). Antioxidant Capacity of Human Milk and Its Association with Vitamins A and E and Fatty Acid Composition. Acta Paediatr..

[92] Krasevec J.M., Jones P.J., Cabrera-Hernandez A., Luisa Mayer D., Connor W.E. (2002). Maternal and Infant Essential Fatty Acid Status in Havana, Cuba. Am. J. Clin. Nutr..

[93] Hørby Jørgensen M., Hernell O., Lund P., Hølmer G., Fleischer Michaelsen K. (1996). Visual Acuity and Erythrocyte Docosahexaenoic Acid Status in Breast-Fed and Formula-Fed Term Infants during the First Four Months of Life. Lipids.

[94] Zou X., Huang J., Jin Q., Guo Z., Liu Y., Cheong L., Xu X., Wang X. (2013). Lipid Composition Analysis of Milk Fats from Different Mammalian Species: Potential for Use as Human Milk Fat Substitutes. J. Agric. Food Chem..

[95] Luukkainen P., Salo M.K., Nikkari T. (1994). Changes in the Fatty Acid Composition of Preterm and Term Human Milk from 1 Week to 6 Months of Lactation. J. Pediatr. Gastroenterol. Nutr..

[96] Maurage C., Guesnet P., Pinault M., Rochettede Lempdes J.-B., Durand G., Antoine J.-M., Couet C. (1998). Effect of Two Types of Fish Oil Supplementation on Plasma and Erythrocyte Phospholipids in Formula-Fed Term Infants. Biol. Neonate.

[97] Pugo-Gunsam P., Guesnet P., Subratty A.H., Rajcoomar D.A., Maurage C., Couet C. (1999). Fatty Acid Composition of White Adipose Tissue and Breast Milk of Mauritian and French Mothers and Erythrocyte Phospholipids of Their Full-Term Breast-Fed Infants. Br. J. Nutr..

[98] Martin J.C., Bougnoux P., Fignon A., Theret V., Antoine J.-M., Lamisse F., Couet C. (1993). Dependence of Human Milk Essential Fatty Acids on Adipose Stores during Lactation. Am. J. Clin. Nutr..

[99] Szabó E., Boehm G., Beermann C., Weyermann M., Brenner H., Rothenbacher D., Decsi T. (2007). Trans Octadecenoic Acid and Trans Octadecadienoic Acid Are Inversely Related to Long-Chain Polyunsaturates in Human Milk: Results of a Large Birth Cohort Study. Am. J. Clin. Nutr..

[100] Genzel-Boroviczény O., Wahle J., Koletzko B. (1997). Fatty Acid Composition of Human Milk during the 1st Month after Term and Preterm Delivery. Eur. J. Pediatr..

[101] Roy S., Dhar P., Ghosh S. (2012). Comparative Evaluation of Essential Fatty Acid Composition of Mothers’ Milk of Some Urban and Suburban Regions of West Bengal, India. Int. J. Food Sci. Nutr..

[102] Haddad I., Mozzon M., Frega N.G. (2012). Trends in Fatty Acids Positional Distribution in Human Colostrum, Transitional, and Mature Milk. Eur. Food Res. Technol..

[103] Scopesi F., Ciangherotti S., Lantieri P.B., Risso D., Bertini I., Campone F., Pedrotti A., Bonacci W., Serra G. (2001). Maternal Dietary PUFAs Intake and Human Milk Content Relationships during the First Month of Lactation. Clin. Nutr..

[104] Nguyen M.T.T., Kim J., Seo N., Lee A.H., Kim Y.K., Jung J.A., Li D., To X.H.M., Huynh K.T.N., van Le T. (2021). Comprehensive Analysis of Fatty Acids in Human Milk of Four Asian Countries. J. Dairy Sci..

[105] Aumeistere L., Ciproviča I., Zavadska D., Andersons J., Volkovs V., Ceļmalniece K. (2019). Impact of Maternal Diet on Human Milk Composition among Lactating Women in Latvia. Medicina.

[106] Khor G.L., Tan S.S., Stoutjesdijk E., Ng K.W.T., Khouw I., Bragt M., Schaafsma A., Dijck-Brouwer D.A.J., Muskiet F.A.J. (2021). Temporal Changes in Breast Milk Fatty Acids Contents: A Case Study of Malay Breastfeeding Women. Nutrients.

[107] Butts C.A., Hedderley D.I., Herath T.D., Paturi G., Glyn-Jones S., Wiens F., Stahl B., Gopal P. (2018). Human Milk Composition and Dietary Intakes of Breastfeeding Women of Different Ethnicity from the Manawatu-Wanganui Region of New Zealand. Nutrients.

[108] van de Heijning B.J.M., Stahl B., Schaart M.W., van der Beek E.M., Rings E.H.H.M., Mearin M.L. (2017). Fatty Acid and Amino Acid Content and Composition of Human Milk in the Course of Lactation. Adv. Pediatr. Res..

[109] Van Beusekom C.M., Nijeboer H.J., van der Veere C.N., Luteyn A.J., Offringa P.J., Muskiet F.A., Boersma E.R. (1993). Indicators of Long Chain Polyunsaturated Fatty Acid Status of Exclusively Breastfed Infants at Delivery and after 20–22 Days. Early Hum. Dev..

[110] Huisman M., van Beusekom C.M., Lanting C.I., Nijeboer H.J., Muskiet F.A., Boersma E.R. (1996). Triglycerides, Fatty Acids, Sterols, Mono- and Disaccharides and Sugar Alcohols in Human Milk and Current Types of Infant Formula Milk. Eur. J. Clin. Nutr..

[111] Bobiński R., Mikulska M., Mojska H., Simon M. (2013). Comparison of the Fatty Acid Composition of Transitional and Mature Milk of Mothers Who Delivered Healthy Full-Term Babies, Preterm Babies and Full-Term Small for Gestational Age Infants. Eur. J. Clin. Nutr..

[112] Szlagatys-Sidorkiewicz A., Martysiak-Zurowska D., Krzykowski G., Zagierski M., Kamińska B. (2013). Maternal Smoking Modulates Fatty Acid Profile of Breast Milk. Acta Paediatr. Int. J. Paediatr..

[113] Ribeiro M., Balcao V., Guimaraes H., Rocha G., Moutinho C., Matos C., Almeida C., Casal S., Guerra A. (2008). Fatty Acid Profile of Human Milk of Portuguese Lactating Women: Prospective Study from the 1st to the 16th Week of Lactation. Ann. Nutr. Metab..

[114] Cruz-Hernandez C., Goeuriot S., Giuffrida F., Thakkar S.K., Destaillats F. (2013). Direct Quantification of Fatty Acids in Human Milk by Gas Chromatography. J. Chromatogr. A.

[115] Sánchez-Hernández S., Esteban-Muñoz A., Giménez-Martínez R., Aguilar-Cordero M.J., Miralles-Buraglia B., Olalla-Herrera M. (2019). A Comparison of Changes in the Fatty Acid Profile of Human Milk of Spanish Lactating Women during the First Month of Lactation Using Gas Chromatography-Mass Spectrometry. A Comparison with Infant Formulas. Nutrients.

[116] Moltó-Puigmartí C., Castellote A.I., Carbonell-Estrany X., López-Sabater M.C. (2011). Differences in Fat Content and Fatty Acid Proportions among Colostrum, Transitional, and Mature Milk from Women Delivering Very Preterm, Preterm, and Term Infants. Clin. Nutr..

[117] Sala-Vila A., Castellote-Bargalló A.I., Rodriguez-Palmero M., Campoy, López-Sabater M.C. (2005). Lipid Composition in Human Breast Milk from Granada (Spain): Changes during Lactation. Nutrition.

[118] Sala-Vila A., Campoy C., Castellote A.I., Garrido F., Rivero M., Rodríguez-Palmero M., López-Sabater M.C. (2006). Influence of Dietary Source of Docosahexaenoic and Arachidonic Acids on Their Incorporation into Membrane Phospholipids of Red Blood Cells in Term Infants. Prostaglandins Leukot Essent Fat. Acids.

[119] López-López A., López-Sabater M.C., Campoy-Folgoso C., Rivero-Urgell M., Castellote-Bargalló A.I. (2002). Fatty Acid and Sn-2 Fatty Acid Composition in Human Milk from Granada (Spain) and in Infant Formulas. Eur. J. Clin. Nutr..

[120] Sala-Vila A., Castellote A.I., Campoy C., Rivero M., Rodriguez-Palmero M., López-Sabater M.C. (2004). The Source of Long-Chain PUFA in Formula Supplements Does Not Affect the Fatty Acid Composition of Plasma Lipids in Full-Term Infants. J. Nutr..

[121] Barreiro R., Díaz-Bao M., Cepeda A., Regal P., Fente C.A. (2018). Fatty Acid Composition of Breast Milk in Galicia (NW Spain): A Cross-Country Comparison. Prostaglandins Leukot Essent Fat. Acids.

[122] Rueda R., Ramírez M., García-Salmerón J.L., Maldonado J., Gil G. (1998). Gestational Age and Origin of Human Milk Influence Total Lipid and Fatty Acid Contents. Ann. Nutr. Metab..

[123] Nyuar K.B., Min Y., Dawood M., Abukashawa S., Daak A., Ghebremeskel K. (2013). Regular Consumption of Nile River Fish Could Ameliorate the Low Milk DHA of Southern Sudanese Women Living in Khartoum City Area. Prostaglandins Leukot Essent Fat. Acids.

[124] Storck Lindholm E., Strandvik B., Altman D., Möller A., Palme Kilander C. (2013). Different Fatty Acid Pattern in Breast Milk of Obese Compared to Normal-Weight Mothers. Prostaglandins Leukot Essent Fat. Acids.

[125] Huang H.-L., Chuang L.-T., Li H.-H., Lin C.-P., Glew R.H. (2013). Docosahexaenoic Acid in Maternal and Neonatal Plasma Phospholipids and Milk Lipids of Taiwanese Women in Kinmen: Fatty Acid Composition of Maternal Blood, Neonatal Blood and Breast Milk. Lipids Health Dis..

[126] Wu T.-C., Lau B.-H., Chen P.-H., Wu L.-T., Tang R.-B. (2010). Fatty Acid Composition of Taiwanese Human Milk. J. Chin. Med. Assoc..

[127] Kuipers R.S., Luxwolda M.F., Dijck-Brouwer D.A.J., Muskiet F.A.J. (2012). Fatty Acid Compositions of Preterm and Term Colostrum, Transitional and Mature Milks in a Sub-Saharan Population with High Fish Intakes. Prostaglandins Leukot Essent Fat. Acids.

[128] Samur G., Topcu A., Turan S. (2009). Trans Fatty Acids and Fatty Acid Composition of Mature Breast Milk in Turkish Women and Their Association with Maternal Diet’s. Lipids.

