# Supplementary material for: The Impact of Linoleic Acid on Infant Health in the Absence or Presence of DHA in Infant Formulas
Source: Nutrients. 2023 May 4;15(9):2187. doi: 10.3390/nu15092187 (PMC10180831; doi:10.3390/nu15092187)
Supplement: Supplementary file 1 [file nutrients-15-02187-s001.zip › nutrients-2338437-supplementary.pdf]

Supplemental Table S1: Average PUFA levels (% of total fatty acids) and ratio of LA to ALA in breast milk reported in studies from different countries

Country [Reference]	Sample size	LA	ALA	DHA	LA/ALA
Australia[1]	69	8.5	0.70	0.2	12.1
Bolivia[2]	35	10.2	1.90	0.69	5.4
Brazil[3–5]	88	20.6	1.51	0.21	14.0
Canada [6–8]	1257	13.5	1.86	0.19	7.3
China [9]	2585	21.8	1.68	0.35	13.0
Cuba[10]	52	19.4	0.92	0.43	21.1
Denmark [11,12]	78	10.7	0.98	0.49	12.4
Finland [13]	7	9.9	1.22	0.3	8.1
France[14–16]	54	12.2	0.57	0.18	21.5
Germany [17,18]	743	10.2	0.7	0.2	14.2
India[19]	135	10.8	1.8	0.7	6.1
Italy[20,21]	74	10.5	0.3	0.2	35.4
Korea [22]	254	17.8	1.87	0.5	9.5
Latvia[23]	61	11.0	1.00	0.3	11.0
Malaysia [24]	26	9.8	0.32	0.56	30.6
New Zealand (Maori & Pacific Island) [25]	17	10.4	1.34	0.18	7.8
Mauritius[15]	15	26.9	2.65	0.38	10.2
Netherlands[26–28]	156	13.1	1.1	0.2	12.4
New Zealand [25]	53	10.4	1.25	0.15	8.3
Pakistan[22]	97	9.1	0.43	0.23	21.2
Poland[29,30]	190	10.0	1.1	0.3	9.5
Portugal [31]	62	16.8	0.7	0.4	24.7
Singapore [32]	50	15.2	1.12	0.63	13.6
Spain[33–40]	152	15.4	0.6	0.3	25.3
Sudan [41]	32	14.7	0.28	0.1	52.5
Sweden [42]	19	10.7	1.47	0.37	7.3
Taiwan [43,44]	78	23.0	1.6	0.9	14.3
Tanzania [45]	34	15.8	0.52	0.53	30.4
Turkey[17,46]	79	21.2	0.6	0.1	34.1
USA [2]	35	18.9	1.58	0.16	12.0
Vietnam [22]	92	17.1	1.17	0.48	14.6

References

1. Mitoulas, L.R.; Gurrin, L.C.; Doherty, D.A.; Sherriff, J.L.; Hartmann, P.E. Infant Intake of Fatty Acids from Human Milk over the First Year of Lactation. *British Journal of Nutrition* **2003**, *90*, 979–986, doi:10.1079/bjn2003979.

2. Martin, M.A.; Lassek, W.D.; Gaulin, S.J.C.; Evans, R.W.; Woo, J.G.; Geraghty, S.R.; Davidson, B.S.; Morrow, A.L.; Kaplan, H.S.; Gurven, M.D. Fatty Acid Composition in the Mature Milk of Bolivian Forager-Horticulturalists: Controlled Comparisons with a US Sample. *Matern Child Nutr* **2012**, *8*, 404–418, doi:10.1111/j.1740-8709.2012.00412.x.

3. Berenhauser, A.C.; Pinheiro Do Prado, A.C.; da Silva, R.C.; Gioielli, L.A.; Block, J.M. Fatty Acid Composition in Preterm and Term Breast Milk. *Int J Food Sci Nutr* **2012**, *63*, 318–325, doi:10.3109/09637486.2011.627843.

4. Nishimura, R.Y.; de Castro, G.S.F.; Jordão, A.A.; Sartorelli, D.S. Breast Milk Fatty Acid Composition of Women Living Far from the Coastal Area in Brazil. *J Pediatr (Rio J)* **2013**, *89*, 263–268, doi:10.1016/j.jped.2012.11.007.

5. Patin, R. v; Vítolo, M.R.; Valverde, M.A.; Carvalho, P.O.; Pastore, G.M.; Ancona Lopez, F. The Influence of Sardine Consumption on the Omega-3 Fatty Acid Content of Mature Human Milk. **2006**, doi:10.2223/JPED.1439.

6. Innis, S.M.; King, D.J. Trans Fatty Acids in Human Milk Are Inversely Associated with Concentrations of Essential All-Cis n-6 and n-3 Fatty Acids and Determine Trans, but Not n-6 and n-3, Fatty Acids in Plasma Lipids of Breast-Fed Infants. *American Journal of Clinical Nutrition* **1999**, *70*, 383–390, doi:10.1093/ajcn/70.3.383.

7. Tijerina-Sáenz, S.; Innis, S.M.; Kitts, DD. Antioxidant Capacity of Human Milk and Its Association with Vitamins A and E and Fatty Acid Composition. *Acta Paediatr* **2009**, *98*, 1793–1798, doi:10.1111/J.1651-2227.2009.01437.X.

8. Miliku, K.; Duan, Q.L.; Moraes, T.J.; Becker, A.B.; Mandhane, P.J.; Turvey, S.E.; Lefebvre, D.L.; Sears, M.R.; Subbarao, P.; Field, C.J.; et al. Human Milk Fatty Acid Composition Is Associated with Dietary, Genetic, Sociodemographic, and Environmental Factors in the CHILD Cohort Study. *American Journal of Clinical Nutrition* **2019**, *110*, 1370–1383, doi:10.1093/ajcn/nqz229.

9. Sun, H.; Ren, Q.; Zhao, X.; Tian, Y.; Pan, J.; Wei, Q.; Li, Y.; Chen, Y.; Zhang, H.; Zhang, W.; et al. Regional Similarities and Differences in Mature Human Milk Fatty Acids in Chinese Population: A Systematic Review. *Prostaglandins Leukot Essent Fatty Acids* 2020, *162*.

10. Krasevec, J.M.; Jones, P.J.; Cabrera-Hernandez, A.; Luisa Mayer, D.; Connor, W.E. Maternal and Infant Essential Fatty Acid Status in Havana, Cuba. *American Journal of Clinical Nutrition* **2002**, *76*, 834–844, doi:10.1093/ajcn/76.4.834.

11. Hørby Jørgensen, M.; Hernell, O.; Lund, P.; Hølmer, G.; Fleischer Michaelsen, K. Visual Acuity and Erythrocyte Docosahexaenoic Acid Status in Breast-Fed and Formula-Fed Term Infants during the First Four Months of Life. *Lipids* **1996**, *31*, 99–105, doi:10.1007/BF02522418.

12. Zou, X.; Huang, J.; Jin, Q.; Guo, Z.; Liu, Y.; Cheong, L.; Xu, X.; Wang, X. Lipid Composition Analysis of Milk Fats from Different Mammalian Species: Potential for Use as Human Milk Fat Substitutes. *J Agric Food Chem* **2013**, *61*, 7070–7080, doi:10.1021/jf401452y.

13. Luukkainen, P.; Salo, M.K.; Nikkari, T. Changes in the Fatty Acid Composition of Preterm and Term Human Milk from 1 Week to 6 Months of Lactation. *J Pediatr Gastroenterol Nutr* **1994**, *18*, 355–360, doi:10.1097/00005176-199404000-00018.

14. Maurage, C.; Guesnet, P.; Pinault, M.; Rochettede Lempdes, J.-B.; Durand, G.; Antoine, J.-M.; Couet, C. Effect of Two Types of Fish Oil Supplementation on Plasma and Erythrocyte Phospholipids in Formula-Fed Term Infants. *Biol Neonate* **1998**, *74*, 416–429, doi:10.1159/000014063.

15. Pugo-Gunsam, P.; Guesnet, P.; Subratty, A.H.; Rajcoomar, D.A.; Maurage, C.; Couet, C. Fatty Acid Composition of White Adipose Tissue and Breast Milk of Mauritian and French Mothers and Erythrocyte Phospholipids of Their Full-Term Breast-Fed Infants. *British Journal of Nutrition* **1999**, *82*, 263–271, doi:10.1017/s0007114599001464.

16. Martin, J.C.; Bougnoux, P.; Fignon, A.; Theret, V.; Antoine, J.-M.; Lamisse, F.; Couet, C. Dependence of Human Milk Essential Fatty Acids on Adipose Stores during Lactation. *Am J Clin Nutr* **1993**, *58*, 653–659, doi:10.1093/AJCN/58.5.653.

17. Szabó, E.; Boehm,G.; Beermann, C.; Weyermann, M.; Brenner, H.; Rothenbacher, D.; Decsi, T. Trans Octadecenoic Acid and Trans Octadecadienoic Acid Are Inversely Related to Long-Chain Polyunsaturates in Human Milk: Results of a Large Birth Cohort Study. *Am J Clin Nutr* **2007**, *85*, 1320–1326, doi:10.1093/AJCN/85.5.1320.

18. Genzel-Boroviczény, O.; Wahle, J.; Koletzko, B. Fatty Acid Composition of Human Milk during the 1st Month after Term and Preterm Delivery. *Eur J Pediatr* **1997**, *156*, 143–147, doi:10.1007/s004310050573.

19. Roy, S.; Dhar, P.; Ghosh, S. Comparative Evaluation of Essential Fatty Acid Composition of Mothers' Milk of Some Urban and Suburban Regions of West Bengal, India. *Int J Food Sci Nutr* **2012**, *63*, 895–901, doi:10.3109/09637486.2012.683778.

20. Haddad, I.; Mozzon, M.; Frega, N.G. Trends in Fatty Acids Positional Distribution in Human Colostrum, Transitional, and Mature Milk. *European Food Research and Technology* **2012**, *2*, 325–332, doi:10.1007/S00217-012-1759-Y.

21. Scopesi, F.; Ciangherotti, S.; Lantieri, P.B.; Risso, D.; Bertini, I.; Campone, F.; Pedrotti, A.; Bonacci, W.; Serra, G. Maternal Dietary PUFAs Intake and Human Milk Content Relationships during the First Month of Lactation. *Clinical Nutrition* **2001**, *20*, 393–397, doi:10.1054/clnu.2001.0464.

22. Nguyen, M.T.T.; Kim, J.; Seo, N.; Lee, A.H.; Kim, Y.K.; Jung, J.A.; Li, D.; To, X.H.M.; Huynh, K.T.N.; van Le, T.; et al. Comprehensive Analysis of Fatty Acids in Human Milk of Four Asian Countries. *J Dairy Sci* **2021**, *104*, 6496–6507, doi:10.3168/jds.2020-18184.

23. Aumeistere, L.; Ciproviča, I.; Zavadska, D.; Andersons, J.; Volkovs, V.; Ceļmalniece, K. Impact of Maternal Diet on Human Milk Composition among Lactating Women in Latvia. *Medicina (Lithuania)* **2019**, *55*, doi:10.3390/medicina55050173.

24. Khor, G.L.; Tan, S.S.; Stoutjesdijk, E.; Ng, K.W.T.; Khouw, I.; Bragt, M.; Schaafsma, A.; Dijck-Brouwer, D.A.J.; Muskiet, F.A.J. Temporal Changes in Breast Milk Fatty Acids Contents: A Case Study of Malay Breastfeeding Women. *Nutrients* **2021**, *13*, 1–13, doi:10.3390/nu13010101.

25. Butts, C.A.; Hedderley, D.I.; Herath, T.D.; Paturi, G.; Glyn-Jones, S.; Wiens, F.; Stahl, B.; Gopal, P. Human Milk Composition and Dietary Intakes of Breastfeeding Women of Different Ethnicity from the Manawatu-Wanganui Region of New Zealand. *Nutrients* **2018**, *10*, doi:10.3390/nu10091231.

26. Heijning, B.J.M. van de; Stahl, B.; Schaart, M.W.; Beek, E.M. van der; Rings, E.H.H.M.; Mearin, M.L. Fatty Acid and Amino Acid Content and Composition of Human Milk in the Course of Lactation. *Adv Pediatr Res* **2017**, *4*, 2–15, doi:10.12715/apr.2017.4.16.

27. Van Beusekom, C.M.; Nijeboer, H.J.; van der Veere, C.N.; Luteyn, A.J.; Offringa, P.J.; Muskiet, F.A.; Boersma, E.R.. Indicators of Long Chain Polyunsaturated Fatty Acid Status of Exclusively Breastfed Infants at Delivery and after 20-22 Days. *Early Hum Dev* **1993**, *32*, 207–218, doi:10.1016/0378-3782(93)90013-K.

28. Huisman, M.; Beusekom, C.M. van; Lanting, C.I.; Nijeboer, H.J.; Muskiet, F.A.; Boersma, E.R. Triglycerides, Fatty Acids, Sterols, Mono- and Disaccharides and Sugar Alcohols in Human Milk and Current Types of Infant Formula Milk. *Eur J Clin Nutr* **1996**, *50*, 255–260.

29. Bobiński, R.; Mikulska, M.; Mojska, H.; Simon, M. Comparison of the Fatty Acid Composition of Transitional and Mature Milk of Mothers Who Delivered Healthy Full-Term Babies, Preterm Babies and Full-Term Small for Gestational Age Infants. *Eur J Clin Nutr* **2013**, *67*, 966–971, doi:10.1038/ejcn.2013.96.

30. Szlagatys-Sidorkiewicz, A.; Martysiak-Zurowska, D.; Krzykowski, G.; Zagierski, M.; Kamińska, B. Maternal Smoking Modulates Fatty Acid Profile of Breast Milk. *Acta Paediatrica, International Journal of Paediatrics* **2013**, *102*, doi:10.1111/apa.12276.

31. Ribeiro, M.; Balcao, V.; Guimaraes, H.; Rocha, G.; Moutinho, C.; Matos, C.; Almeida, C.; Casal, S.;Guerra, A. Fatty Acid Profile of Human Milk of Portuguese Lactating Women: Prospective Study from the 1st to the 16th Week of Lactation. *Ann Nutr Metab* **2008**, *53*, 50–56, doi:10.1159/000156597.

32. Cruz-Hernandez, C.; Goeuriot, S.; Giuffrida, F.; Thakkar, S.K.; Destaillats, F. Direct Quantification of Fatty Acids in Human Milk by Gas Chromatography. *J Chromatogr A* **2013**, *1284*, 174–179, doi:10.1016/J.CHROMA.2013.01.094.

33. Sánchez-Hernández, S.; Esteban-Muñoz, A.; Giménez-Martínez, R.; Aguilar-Cordero, M.J.; Miralles-Buraglia, B.; Olalla-Herrera, M. A Comparison of Changes in the Fatty Acid Profile of Human Milk of Spanish Lactating Women during the First Month of Lactation Using Gas Chromatography-Mass Spectrometry. A Comparison with Infant Formulas. *Nutrients* **2019**, *11*, doi:10.3390/nu11123055.

34. Moltó-Puigmartí, C.; Castellote, A.I.; Carbonell-Estrany, X.; López-Sabater, M.C. Differences in Fat Content and Fatty Acid Proportions among Colostrum, Transitional, and Mature Milk from Women Delivering Very Preterm, Preterm, and Term Infants. *Clinical Nutrition* **2011**, *30*, 116–123, doi:10.1016/J.CLNU.2010.07.013.

35. Sala-Vila, A.; Castellote-Bargalló, A.I.; Rodriguez-Palmero, M.; Campoy; López-Sabater, M.C. Lipid Composition in Human Breast Milk from Granada (Spain): Changes during Lactation. *Nutrition* **2005**, *21*, 467–473, doi:10.1016/J.NUT.2004.08.020.

36. Sala-Vila, A.; Campoy, C.; Castellote, A.I.; Garrido, F.; Rivero, M.; Rodríguez-Palmero, M.; López-Sabater, M.C. Influence of Dietary Source of Docosahexaenoic and Arachidonic Acids on

Their Incorporation into Membrane Phospholipids of Red Blood Cells in Term Infants. *Prostaglandins Leukot Essent Fatty Acids* **2006**, *74*, 143–148, doi:10.1016/J.PLEFA.2005.10.003.

37. López-López, A.; López-Sabater, M.C.; Campoy-Folgoso, C.; Rivero-Urgell, M.; Castellote-Bargalló, A.I. Fatty Acid and Sn-2 Fatty Acid Composition in Human Milk from Granada (Spain) and in Infant Formulas. *Eur J Clin Nutr* **2002**, *56*, 1242–1254, doi:10.1038/sj.ejcn.1601470.

38. Sala-Vila, A.; Castellote, A.I.; Campoy, C.; Rivero, M.; Rodriguez-Palmero, M.; López-Sabater, M.C. The Source of Long-Chain PUFA in Formula Supplements Does Not Affect the Fatty Acid Composition of Plasma Lipids in Full-Term Infants. *J Nutr* **2004**, *134*, 868–873.

39. Barreiro, R.; Díaz-Bao, M.; Cepeda, A.; Regal, P.; Fente, C.A. Fatty Acid Composition of Breast Milk in Galicia (NW Spain): A Cross-Country Comparison. *Prostaglandins Leukot Essent Fatty Acids* **2018**, *135*, 102–114, doi:10.1016/j.plefa.2018.06.002.

40. Rueda, R.; Ramírez, M.; García-Salmerón, J.L.; Maldonado, J.; Gil, G. Gestational Age and Origin of Human Milk Influence Total Lipid and Fatty Acid Contents. *Ann Nutr Metab* **1998**, *42*, 12–22, doi:10.1159/000012713.

41. Nyuar, K.B.; Min, Y.; Dawood, M.; Abukashawa, S.; Daak, A.; Ghebremeskel, K. Regular Consumption of Nile River Fish Could Ameliorate the Low Milk DHA of Southern Sudanese Women Living in Khartoum City Area. *Prostaglandins Leukot Essent Fatty Acids* **2013**, *89*, 65–69, doi:10.1016/J.PLEFA.2013.04.007.

42. Storck Lindholm, E.; Strandvik, B.; Altman, D.; Möller, A.; Palme Kilander, C. Different Fatty Acid Pattern in Breast Milk of Obese Compared to Normal-Weight Mothers. *Prostaglandins Leukot Essent Fatty Acids* **2013**, *88*, 211–217, doi:10.1016/J.PLEFA.2012.11.007.

43. Huang, H.-L.; Chuang, L.-T.; Li, H.-H.; Lin, C.-P.; Glew, R.H.. Docosahexaenoic Acid in Maternal and Neonatal Plasma Phospholipids and Milk Lipids of Taiwanese Women in Kinmen: Fatty Acid Composition of Maternal Blood, Neonatal Blood and Breast Milk. *Lipids Health Dis* **2013**, *12*, doi:10.1186/1476-511X-12-27.

44. Wu, T.-C.; Lau, B.-H.; Chen, P.-H.; Wu, L.-T.; Tang, R.-B. Fatty Acid Composition of Taiwanese Human Milk. *J Chin Med Assoc* **2010**, *73*, 581–588, doi:10.1016/S1726-4901(10)70127-1.

45. Kuipers, R.S.; Luxwolda, M.F.; Dijck-Brouwer, D.A.J.; Muskiet, F.A.J. Fatty Acid Compositions of Preterm and Term Colostrum, Transitional and Mature Milks in a Sub-Saharan Population with High Fish Intakes. *Prostaglandins Leukot Essent Fatty Acids* **2012**, *86*, 201–207, doi:10.1016/j.plefa.2012.02.006.

46. Samur, G.; Topcu, A.; Turan, S. Trans Fatty Acids and Fatty Acid Composition of Mature Breast Milk in Turkish Women and Their Association with Maternal Diet's. *Lipids* **2009**, *44*, 405–413, doi:10.1007/s11745-009-3293-7.
